# Biological and Molecular Components for Genetically Engineering Biosensors in Plants

**DOI:** 10.34133/2022/9863496

**Published:** 2022-11-09

**Authors:** Yang Liu, Guoliang Yuan, Md Mahmudul Hassan, Paul E. Abraham, Julie C. Mitchell, Daniel Jacobson, Gerald A. Tuskan, Arjun Khakhar, June Medford, Cheng Zhao, Chang-Jun Liu, Carrie A. Eckert, Mitchel J. Doktycz, Timothy J. Tschaplinski, Xiaohan Yang

**Affiliations:** ^1^Biosciences Division, Oak Ridge National Laboratory, Oak Ridge, Tennessee 37831, USA; ^2^The Center for Bioenergy Innovation, Oak Ridge National Laboratory, Oak Ridge, Tennessee 37831, USA; ^3^Department of Genetics and Plant Breeding, Patuakhali Science and Technology University, Dumki, Patuakhali, 8602, Bangladesh; ^4^Department of Biology, Colorado State University, Fort Collins, Colorado 80523, USA; ^5^Shenzhen Branch, Guangdong Laboratory for Lingnan Modern Agriculture, Key Laboratory of Synthetic Biology, Ministry of Agriculture and Rural Affairs, Agricultural Genomics Institute at Shenzhen, Chinese Academy of Agricultural Sciences, Shenzhen 518120, China; ^6^Biology Department, Brookhaven National Laboratory, Upton, New York 11973, USA

## Abstract

Plants adapt to their changing environments by sensing and responding to physical, biological, and chemical stimuli. Due to their sessile lifestyles, plants experience a vast array of external stimuli and selectively perceive and respond to specific signals. By repurposing the logic circuitry and biological and molecular components used by plants in nature, genetically encoded plant-based biosensors (GEPBs) have been developed by directing signal recognition mechanisms into carefully assembled outcomes that are easily detected. GEPBs allow for *in vivo* monitoring of biological processes in plants to facilitate basic studies of plant growth and development. GEPBs are also useful for environmental monitoring, plant abiotic and biotic stress management, and accelerating design-build-test-learn cycles of plant bioengineering. With the advent of synthetic biology, biological and molecular components derived from alternate natural organisms (e.g., microbes) and/or *de novo* parts have been used to build GEPBs. In this review, we summarize the framework for engineering different types of GEPBs. We then highlight representative validated biological components for building plant-based biosensors, along with various applications of plant-based biosensors in basic and applied plant science research. Finally, we discuss challenges and strategies for the identification and design of biological components for plant-based biosensors.

## 1. Introduction

Environmental perturbations and threats jeopardize many ecosystems on Earth. Real-time monitoring of biological processes, environmental threats, and ecosystem responses, with informative spatial and temporal resolution, remains a challenge, as traditional monitoring strategies are typically based on laboratory analysis of samples collected at specific time points and locations [1–3]. Biosensors offer an alternative to traditional destructive sampling schemes. Molecules, organisms, or devices in a biological context that sense specific stimuli or molecules and convert such signals into a quantitative or qualitative indicator can function as biosensors [4]. With the power of fluorescent proteins and other visible reporters, genetically encoded visible biosensors provide promising tools for large-scale environmental monitoring, high-resolution live-cell imaging, and so on.

Plants are natural biosensors, responding to various environmental stimuli and generating real-time signals for inter- and intracellular communication [5]. Native, signal-responsive promoters, signaling motifs (e.g. nuclear export signals (NESs)), and short amino acids sequences (i.e., degrons), have been used as sensory components for engineering genetically encoded plant-based biosensors (GEPBs) [6–8]. While GEPBs allow scientists to monitor molecular events, cell activities, and metabolic pathways in real-time, they also offer multiple desirable features for environmental monitoring. First, the integrated aboveground leaves and belowground root system allow plants to not only accept signals in the air, but to also sense the dynamic signals from a broad region of the soil [9]. Second, engineering biosensor in perennial plants species allows for cost-effective continuous monitoring of environmental conditions [9]. Third, with visible plant-based biosensors responsive to environmental factors, cumbersome sampling and laboratory analysis can be avoided [10].

An ideal plant-based biosensor should respond to specific stimuli and produce a quantifiable signal without disturbing the endogenous plant system. It has been difficult to develop such plant biosensor systems based solely on endogenous components due to the complicated crosstalk among different biological pathways. For instance, promoter-reporter systems derived from hormone signaling pathways (e.g., *DR5* and *TCS::GFP*) can be affected by native pathways [11]. Furthermore, many protein-degradation-based sensors (e.g., DII-VENUS and GFP-DELLA) are irreversible, and thus once triggered miss transient or dynamic signal changes [11, 12]. Hence, there is a need for new strategies to accelerate plant biosensor engineering.

Plant biosystem design or plant synthetic biology is an emerging and quickly advancing field, which aims to design elite plants with desirable traits [4, 13]. Synthetic biology potentially offers interchangeable components, standardized genetic circuit design, and Design-Build-Test-Learn (DBTL) cycles for rapidly developing plant-based biosensors [14]. The application of fluorescence proteins (FPs) in biosensor design is a good example demonstrating how synthetic biology utilizes universal components for building biosensors in various organisms. Standardization is important for plant biosystem design. Currently, there are no standard pipelines for choosing components to design and build genetic circuits as plant-based biosensors. In this review, we categorize plant-based biosensors based on the mode of action and list representative validated biological and molecular components for engineering each type of biosensor with the goal of proposing a standard roadmap for building genetically encoded plant-based biosensors. In addition, we discuss how to identify and design new biological components for bioengineering to expand the applications of plant-based biosensors.

## 2. Framework for Engineering Genetically Encoded Plant-Based Biosensors

**Figure 1:**
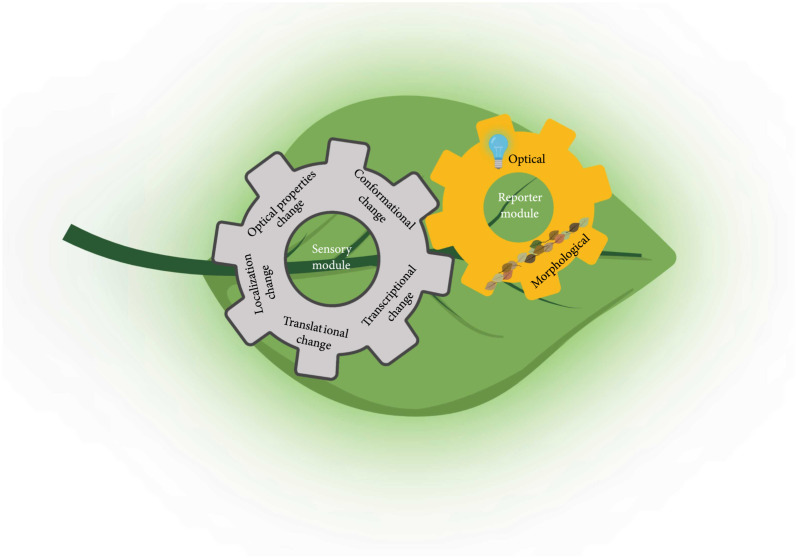
Conceptual framework of genetically encoded plant-based biosensors (GEPBs) design. GEPBs contain a Sensory Module and a Reporter Module. Five different mechanisms of Sensory Modules are presented in the gray gear, and two types of Reporters Module are presented in the yellow gear. The Sensory Module detects the environmental, chemical, or internal stimuli, and the Reporter Module provides the detectable signal.

Standardized components and frameworks are the first step towards a universal roadmap for building GEPBs. In general, GEPBs consist of a sensory element coupled to visible reporter proteins [11] (Figure 1). Based on whether biosensors rely on the endogenous cellular processes, they can be categorized into two types: direct and indirect biosensors. Direct biosensors typically rely on fluorescence proteins (FPs) whose properties are altered by the sensing activities or interaction with analytes, while indirect biosensors consist of cellular components involved in transcription, posttranslational modification, or translocation.

**Figure 2:**
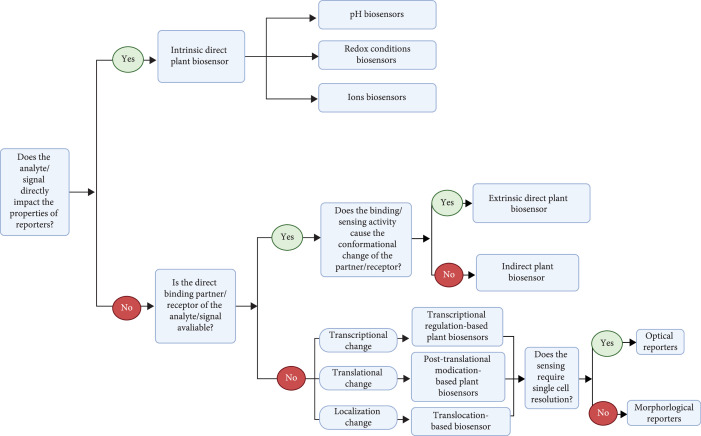
A dichotomous decision tree for the selection of appropriate genetically encoded plant-based biosensors.

Each biosensor type has advantages and limitations. Direct biosensors are a basic bioimaging tool with straightforward design principles, high sensitivity, and a wide range of resolution. Such direct biosensors produce reversible readouts that can reflect the real-time change of cellular processes. Alternatively, indirect biosensors rely on cellular process, e.g., transcription or translation, adding regulatable layers of output that can be fine-tuned. The genetic circuit design for indirect biosensors can also be used to produce metabolites or proteins as reporters. Hence, indirect biosensors offer more advanced tools with flexible designs that expand the biosensor toolbox to benefit plant biosystem design. However, because the output of indirect biosensors is usually irreversible, it is difficult to use indirect biosensors to monitor real-time dynamic cellular processes. In this section, strategies for designing different types of biosensors will be discussed and a decision map for applying different types of plant-based biosensors is illustrated in Figure 2.

### 2.1. Direct Plant Biosensors

Direct biosensors require no additional sensory module or only one sensory module directly interacting with the analytes. There are two types of direct biosensors, intrinsic and extrinsic direct biosensors. The intrinsic direct biosensors harbor reporters sensitive to the cellular environment, whereas the extrinsic direct biosensors contain a sensory module whose structural change, causing a change in distance/orientation of two reporters, leading to signal changes [15]. Because the structural change is always reversible, direct biosensors allow detection of dynamic processes [16].

#### 2.1.1. Intrinsic Direct Plant Biosensors

**Figure 3:**
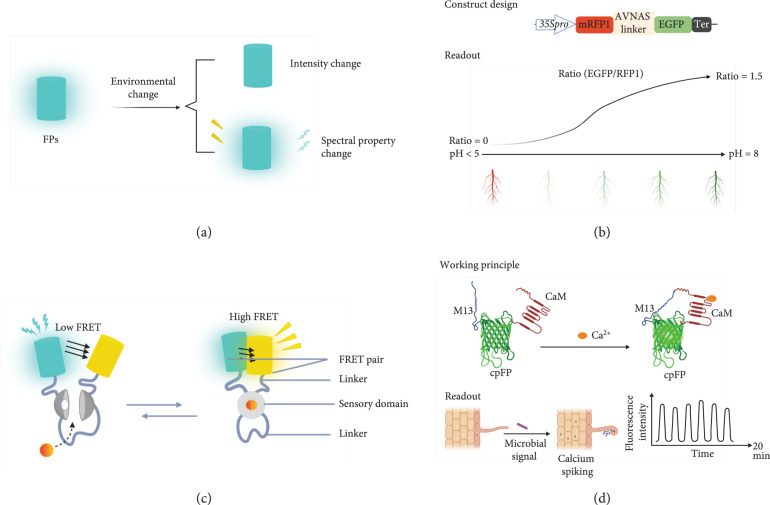
Design of direct plant biosensors. (a) Working principle for intrinsic direct plant biosensors where florescent proteins (FP) respond to an environmental change and induce a spectral change in the FP. (b) Illustration of the construct design and readout of pHusion in plants. Figures are redrawn from the publication of pHusion [51]. Pro indicates promoter and ter represents terminator. (c) Working principle for extrinsic direct plant biosensors, where the conformational change of the sensory module caused by binding activity leads to the FRET change. Figure is designed based on the strategy of FRET-based biosensors. (d) Illustration of working principle and application of G-GECO in plants. Figures are redesigned from the results in *Populus* [68].

FPs can be readily detected by fluorescence microscopy without adding fluorescent dyes, making them useful visible reporters for noninvasive imaging (Figures 3(a) and 3(b)). For example, a phenol group on the chromophore allows pH to affect the optical properties of most green fluorescent protein (GFP) variants [17]. The structural basis of wild-type GFP (wtGFP) and the chromophore of GFP, including three consecutive amino acids S65, Y66, and G67 and residues responsible for florescence, have been identified [17]. Here, the excitation property of FPs is related to the ionization state of the chromophore, whose equilibrium is controlled by a hydrogen bond network that permits proton transfer to the neighboring amino acids when the chromophore is excited [18, 19]. This understanding has ushered in the opportunity for expanded FP engineering.

Most intrinsic direct biosensors were developed through targeted mutagenesis or random mutagenesis of the chromophore of FPs [20]. The abundance of available FPs facilitates potential applications for a wide variety of biosensors for pH, ion concentration, and redox conditions within plant cells [18, 21–24]. Some FPs emit different fluorescence intensity in various cellular environments, while other modified FPs have bimodal excitation/emission spectra, where the excitation/emission ratio reflects the change of cellular environmental conditions [25, 26]. This type of biosensor solely relies on the spectral properties of FPs, so the intrinsic direct biosensors require the least number of components compared with other types of biosensors. The validated intensity-based and ratiometric intrinsic direct biosensors will be discussed in Section 3.

#### 2.1.2. Extrinsic Direct Plant Biosensors

Different FPs can also be fused to a sensory module to enable the use of a fluorescence resonance energy transfer (FRET) signal as a readout and is known as a FRET-based biosensor (Figure 3(c)). In this circumstance, any confirmational change in the sensory module will alter the distance/orientation of the two FPs and thereby modify the FRET efficiency [27]. Based on a similar principle, using different luciferase variants, bioluminescence resonance energy transfer (BRET)-based biosensors have been developed for imaging protein interactions in plant and mammalian cells and tissues [28, 29]. Because readout of FRET-based biosensors is excitation/emission ratio, this type of biosensor is ratiometric (i.e., the output is directly proportional to the input).

The tolerance of GFPs for circular permutations and insertion of entire proteins offers another strategy for building extrinsic direct plant biosensors [30]. The conformational changes in the insert can influence the spectral properties of these circular permutated FPs. Hence, single circularly permutated FPs (cpFPs) can fuse with the sensory module to build extrinsic direct biosensors (Figure 3(d)). The readout of this type of extrinsic direct biosensors can be either ratiometric or intensiometric (i.e., intensity change depending on the design).

Based on a specific design principle, the direct binding partner/receptor for the analyte is necessary for designing extrinsic direct biosensors. Moreover, the structural change induced by the binding/sensing activity is another important factor to be considered for designing extrinsic direct biosensors. Modification of the sensory module can be applied for adjusting the analyte affinity [11]. Extrinsic direct plant biosensors that have been widely used for sensing various analytes will be presented in detail in Section 3.

### 2.2. Indirect Plant Biosensors

Direct binding partner/receptors are not always feasible in biosensor design. Under these circumstances, the downstream cellular processes induced by the binding/sensing activity can be applied as indicators for the signals, known as an indirect biosensor. Indirect biosensors need additional cellular processes to render a signal for a particular analyte or stimulus, and are typically grouped as transcription-based biosensors, posttranslational-based biosensors, and translocation-based biosensors. To build indirect plant-based biosensors, biological parts are usually found within the system under study. Because biological processes are complicated, indirect biosensors typically require a greater number of biological and molecular parts and are more complicated in comparison to direct biosensors.

#### 2.2.1. Transcriptional Regulation-Based Plant Biosensors

**Figure 4:**
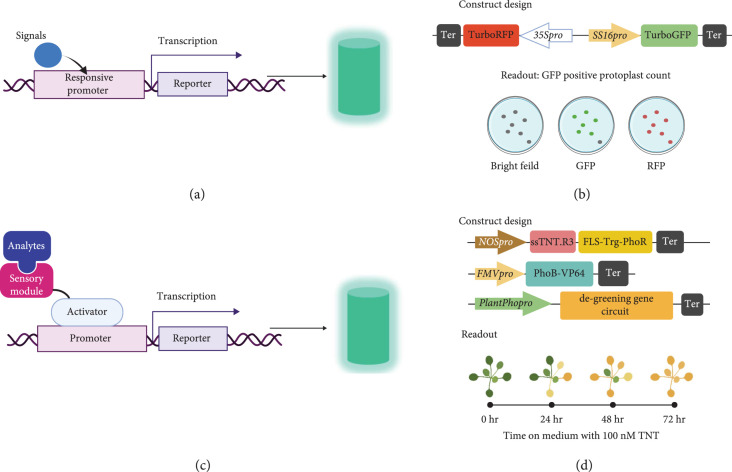
Design of transcriptional regulation-based plant biosensors. (a) Working principle for transcriptional regulation-based biosensors with responsive promoters as the sensory module, where the turquoise cylinder presents an optical signal from a florescent protein. (b) Illustration of the construct design and readout of SS16::GFP biosensor. Figures were redrawn from the results in *Populus* protoplasts [33]. Pro indicates promoter, and ter represents terminator. (c) Working principle for transcriptional regulation-based biosensors with synthetic TFs as the sensory module. (d) Illustration of constructs design and readout of TNT plant-based biosensor. Figures were adopted from phenotypic data of *Arabidopsis* [38].

If the binding/sensing activity causes a transcriptional change in the plant system, transcriptional regulation-based biosensors can be considered. Transcription factors (TFs) and promoters are two main components of plant transcriptional machinery that serve as sensory modules for transcriptional regulation-based biosensor design. There are two types of transcription-based biosensors depending on the sensory module (Figure 4). The first, known as a promoter-reporter system, utilizes promoters as the sensory module that regulates the transcription level of reporters (Figures 4(a) and 4(b)). A native plant gene promoter is the DNA sequence upstream of the transcription start sites (TSS) of a gene. A good example of transcription-based biosensors is *β*-glucuronidase or FPs driven by a synthetic or native hormone-responsive promoter for measuring phytohormone accumulation [31, 32]. However, because of the complex signaling networks within a plant system, applying endogenous plant promoters for sensory modules often results in low and complex patterns of readouts [33]. To enhance the specificity and sensitivity of the promoters, multiple core response elements can be used to create a synthetic promoter. For example, the synthetic DR5 promoter, consisting of 7-9 TGTCTC Aux response element repeats, have been used to drive the expression of different reporters such as *β*-glucuronidase, fluorescent proteins, or luciferase to create a biosensor for auxin [31].

The second type of transcription-based biosensor uses synthetic TFs as the sensory module (Figure 4(c)). Here a TF is fused to a degron or conditionally stable ligand binding domain to construct a responsive synthetic activator/repressor regulated by posttranslational modifications [34, 35]. Although the responsive degrons or conditionally stable ligand binding domains can be directly fused with reporters to design posttranslational regulation-based plant biosensors, the synthetic TFs can amplify the biosensor response and serve as a ligand-dependent controller for gene expression [35].

Endogenous signaling transduction systems (i.e., native plant promoters and TFs) can interfere with transcription-based biosensors. To overcome this concern, several orthogonal regulatory systems from *Saccharomyces* spp., bacteria, and other organisms have been developed for modulating transcription in plants [36, 37]. Likewise, a functional signal transduction system from bacteria has been engineered for plant transcriptional regulation-based biosensor design [38] (Figure 4(d)). The detail design strategies of those orthogonal regulators will be discussed in Section 3.

#### 2.2.2. Posttranslational Modification-Based Plant Biosensors

**Figure 5:**
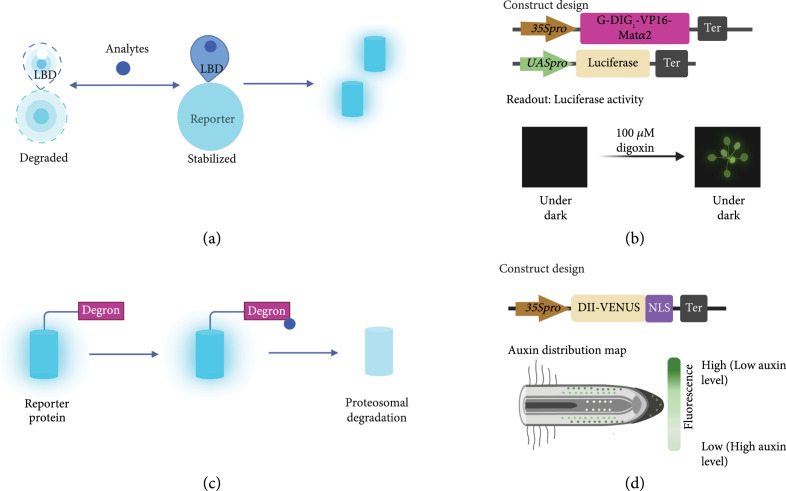
Design of posttranslational modification-based plant biosensors. (a) Working principle for posttranslational modification-based biosensors consisting of a conditionally stable ligand binding domain (LBD) fused with a reporter, where the turquoise cylinder presents an optical signal from a florescent protein. (b) Illustration of constructs design and application of digoxin plant-based biosensor. Figures were redrawn from results in *Arabidopsis* [35]. Pro indicates promoter and ter represents terminator. (c) Working principle for posttranslational modification-based biosensors consisting of a degron motif fused with a reporter. (d) Illustration of constructs design and application of DII-VENUS for mapping Auxin distribution. Figures were redrawn from results in *Arabidopsis* [6]. Pro indicates promoter, and ter represents terminator.

A change in protein level, i.e., an accumulated reporter, has been utilized for biosensor design (Figure 5). Conditionally stable ligand-binding domains (LBDs) linked to reporters have been used as a general strategy to build small molecule biosensors [35]. Such engineered LBDs are degraded by the ubiquitin proteasome system until it binds to the signal molecules and thereby creates sensing/binding activity (Figure 5(a)). Currently, some conditionally stable LBDs have been engineered for sensing small molecules based on computational design [35, 39]. For example, an engineered LBD biosensor for digoxigenin has been applied in plants [35] (Figure 5(b)). A robust computational design is the first step for LBD engineering. Next, high-throughput experimental characterization is required for evaluating the ligand-dependent responses. Fluorescence-activated cell sorting (FACS) is a commonly used strategy. To obtain the ideal LBD-based biosensor, one round of optimization is usually needed. Error-prone PCR and site-saturation mutagenesis are two methods for creating a variable library of LBDs for characterization.

Another strategy is to apply a signal responsive degron as the sensory module coupled with a reporter gene (Figure 5(c)). The binding of the target analyte promotes degradation of the degron that thus yields a signal. A well-known degron-based biosensor DII-VENUS reporter system was developed by fusing the VENUS yellow fluorescent protein in frame to the degron motif of AtIAA28 [6] (Figure 5(d)). Jas9-VENUS has also been developed for mapping local changes in JA levels in *Arabidopsis* roots [40]. Fluorescence levels for these two biosensors are inversely correlated to endogenous phytohormone levels.

#### 2.2.3. Translocation-Based Plant Biosensors

**Figure 6:**
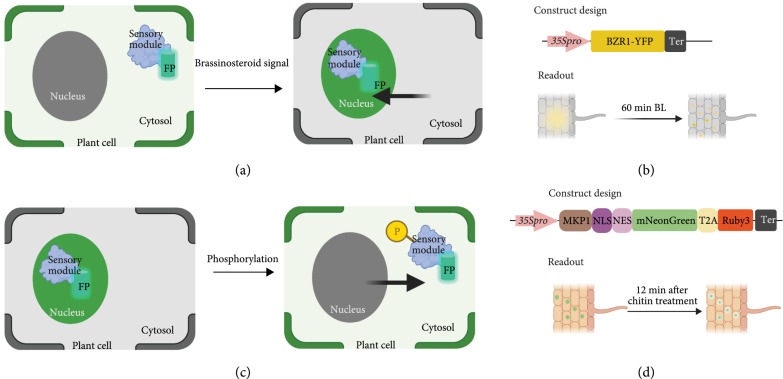
Design of translocation-based biosensors. (a) Illustration of translocation-based biosensors for brassinosteroid signaling pathway. (b) Illustration of the construct design and readout of BZR1-YFP biosensor. Yellow dots represent the accumulation of BZR1-YFP in nucleus, whereas the gradient yellow area represents the cytoplasmic BZR1-YFP signals. Figures were adopted from a study in *Arabidopsis* [42]. Pro indicates promoter, and ter represents terminator. (c) Illustration of translocation-based biosensors for phosphorylation activity. (d) Illustration of the construct design and readout of the KLR-MKP1 biosensor. Solid green spots represent the accumulation of KLR-MKP1 signals in the nucleus, whereas the green circle with gradient color change indicates the decrease fluorescence intensity in the nucleus. Figures were adopted from the results in *Arabidopsis* [96]. Pro indicates promoter, and ter represents terminator.

While transcriptional regulation-based and posttranslational modification-based biosensors monitor the input signal by changes in the abundance of reporters, and translocation-based biosensors utilize spatial location of reporters to measure signal levels. The localization of proteins can be tracked by fusing a FP to a protein of interest (Figure 6). The change in localization of the sensory module with a reporter can be used as a readout to monitor the input signal. This kind of biosensor is an ideal tool for studying protein localization, hormone signaling pathways, and protein phosphorylation levels [11, 27, 41]. For instance, because the brassinosteroid (BR) signaling promotes BRASSINAZOLE RESISTANT 1 (BZR1) nuclear localization, BZR1 fused with YFP can serve as a biosensor for BR signaling in *Arabidopsis* roots and hypocotyls [42] (Figures 6(a) and 6(b)).

Another example of translocation-based biosensors are kinase translocation reporters (KTRs) (Figures 6(c) and 6(d)). KTRs are comprised of an FP fused to a substrate recognition motif for a target kinase, where phosphorylation sites are attached to a negatively phospho-regulated nuclear localization signal (NLS) and a positively phospho-regulated nuclear export signal (NES) [43]. A nonphosphorylated KTR locates within the nucleus via its NLS. Once there, phosphorylation of the NES allows KTR to exit the nucleus, which leads to the conversion of a phosphorylation event into a nucleocytoplasmic shuttling event [41, 44].

Similarly, the intein-mediated conditional protein splicing (CPS) reaction has become an important strategy for protein engineering [45, 46]. The intein-mediated split NLS and NES has also been applied for building translocation-based biosensors [47, 48]. Essentially, the split signal peptide (NLS or NES) is fused to a FP serves as a reporter. Upon the sensing activity, the signal peptide will be reconstituted via intein-mediated protein splicing, resulting in the translocation of signals. Thus far, this strategy has not been applied for building translocation-based biosensors in plants.

Translocation-based biosensors can also be used for detecting gene expression in real time. PP7 and MS2 RNA labeling technologies have been applied to building plant biosensors for gene expression [49]. Nascent RNA labeling with an RNA aptamer such as the MS2 or PP7 recruits FPs fused with an MS2 coat protein or PP7 bacteriophage coat protein to transcriptional loci [49]. The fluorescence intensity of these signals reflects the instantaneous rate of transcription [49].

## 3. Validated Biological Components for Different Types of Plant-Based Biosensors

Based on the framework discussed in Section 2, here biological components including sensory modules (promoter sequences, protein-coding sequences, and noncoding RNAs) and reporters (optical and morphological) are reviewed in the context of validated biological components for different types of plant biosensors. In this section, the application of frameworks mentioned above for designing various plant biosensors will be discussed in the context of further guidance for plant biosensor design.

### 3.1. Validated Biological Components for Direct Plant Biosensors

As mentioned in Section 2.1, FPs have been modified as intrinsic direct biosensors for sensing different analytes and changes of cellular environment. Representative biological components and design illustrations of intrinsic direct plant-based biosensors are listed in Table 1. With an additional sensory module, many extrinsic direct biosensors have been designed to sense direct signal binding activities. Validated sensory modules and design strategies for extrinsic direct plant-based biosensors are listed in Table 2.

**Table 1:** Selected examples of validated biological parts for intrinsic direct plant biosensors. Coding sequences for listed biosensors are provided in supplemental data 1.

Name	Readout	Description	Biosensor target	Tested plant species	References
pHusion	Ratiometric	The tandem concatenation of enhanced green fluorescent protein (EGFP) and monomeric red fluorescent protein (mRFP1)	pH	*Arabidopsis thaliana*	[51]
PE-pHluorin	Intensity	Plant-solubility-modified ecliptic pHluorin	pH	*A. thaliana*	[131]
PR-pHluorin	Ratiometric	Plant-solubility-modified ratiometric pHluorin	pH	*A. thaliana*	[131]
Pt-GFP	Ratiometric	A ratiometric GFP variant from *Ptilosarcus gurneyi*	pH	*A. thaliana*, *Solanum tuberosum*	[132, 133]
Acidin2	Ratiometric	The tandem concatenation of mRFP and tagBFP2	pH	*A. thaliana*	[52]
Acidin3	Ratiometric	The tandem concatenation of mRFP and gamillus	pH	*A. thaliana*	[52]
Acidin4	Ratiometric	The tandem concatenation of mRFP and SYFP2	pH	*A. thaliana*	[52]
Clomeleon	Ratiometric	Cl^-^ indicator (CFP-rTEV-YFP)	Chloride concentration	*A. thaliana*	[55, 56]
roGFP2	Ratiometric	A redox-dependent GFP variant that displays a bimodal excitation spectrum	Redox condition	*A. thaliana*	[61]
BS1	Intensity	A mutated dark GFP with a gRNA targeting the mutated region	CRISPR/Cas9	*A. thaliana*, *Nicotiana benthamiana*, *Populus tremula×alba* 717-1B4, *P. deltoides* WV94	[62]
BS2	Intensity	A mutated dark GFP with a gRNA targeting the mutated region	CRISPR/Cas9-based base editors	*A. thaliana*, *N. benthamiana*, *P. tremula×alba* 717-1B4, *P. deltoides* WV94	[62]

**Table 2:** Selected examples of validated biological parts for extrinsic direct plant biosensors. Coding sequences for listed biosensors are provided in supplemental data 2.

Name	Readout	Description	Biosensor target	Tested plant species	References
G-GECO	Intensity	Genetically encoded calcium indicators with improved single cpGFP for optical imaging (M13-cpGFP-CaM)	Ca^2+^	*Arabidopsis thaliana*, *Populus tremula*×*alba* 717-1B4	[67, 68]
Cameleons	Ratiometric	Genetically encoded calcium indicators with two FPs (CFP/BFP-CaM-M13-GFP/YFP)	Ca^2+^	*A. thaliana*	[134–137]
CALWY	Ratiometric	Genetically encoded zinc indicators with two FPs (cerulean-Atox1-WD4-citrine)	Zn^2+^	*A. thaliana*	[71]
FLIPPi	Ratiometric	Genetically encoded phosphate indicators with two FPs (eCFP-PiBP-eYFP)	Pi	*A. thaliana*	[73]
cpFLIPPi	Ratiometric	Genetically encoded phosphate indicators with two FPs (eCFP-PiBP-cpVenus)	Pi	*A. thaliana*	[73]
ABSCUS1	Ratiometric	Genetically encoded ABA indicator with two FPs (edCerulean-PYL1-ABI1aid-edCitrine)	ABA	*A. thaliana*	[74]
AuxSen	Ratiometric	Genetically encoded IAA indicator with two FPs (aquamarine-TrpR-mNeonGreen)	IAA	*A. thaliana*	[12]
SED1	Ratiometric	Genetically encoded osmotic stress biosensor with two FPs (mCerulean3-AtLEA4-5-citrine)	Osmotic stress	*A. thaliana*	[75]

#### 3.1.1. Validated Components for Intrinsic Direct Plant Biosensors

Taking advantage of pH-dependent excitation and emission properties of FPs, a single FP can be used as an intrinsic direct plant biosensor for measuring pH within plant cells. An example is the ratiometric phGFP that has been expressed in *Arabidopsis* for monitoring intracellular pH changes. This direct biosensor exhibited a dynamic range of 410 nm/470 nm excitation ratio for pH from 5.5 to 7.5 [50]. Tandem fusion of different FPs has also been applied for intrinsic direct plant biosensor design. The pH sensor named ‘pHusion’ consists of a monomeric red fluorescent protein (mRFP1) and an enhanced GFP [51]. Currently, three new ratiometric biosensors composed of two FPs in tandem have been generated for measuring a pH range of 3-8 within the plant cell apoplast [52].

Beyond the intrinsic pH sensitivity, ions near chromophores impact the spectral properties of FPs, which allows FPs to be engineered to measure the concentrations of various chemical ions within plant cells. YFP and its variants have been constructed as a halide biosensor, where fluorescence decreased with increasing concentrations of chloride or nitrate [53, 54]. A ratiometric optical indicator for chloride concentration has been constructed by fusing the chloride-sensitive YFP and chloride insensitive cyan fluorescent protein [55]. This biosensor can be applied for the monitoring salt stress [56]. Engineering the metal binding sites of the Av-GFP resulted in a highly specific direct biosensor, eGFP20C, for monitoring mercury uptake [57]. Additional intrinsic direct biosensors can be developed by engineering the potential metal binding sites of FPs.

Another important application of intrinsic plant biosensors is real-time monitoring of plant cell redox conditions and energy physiology. In plants, redox reactions are involved in photosynthesis, respiration, and many other plant energy metabolism pathways [18]. Reactive oxygen species (ROS) are key signals in plant development and stress responses [18]. Genetically encoded intrinsic direct plant biosensors allow monitoring the dynamics of redox conditions and energy metabolites at high spatial resolution, which provides detailed insights into plant physiology [18, 58]. Structural changes in the FP ß-barrel, redox-sensitive YFP (rxYFP), and redox-sensitive GFP (roGFP) have been engineered and used as intrinsic plant biosensors [59]. Among these, roGFP2 is the most commonly used because of its photostability, pH-insensitivity, and good signal-to-noise ratio [60, 61].

FPs can also be modified to detect the CRISPR machinery in plants. Intentionally inactivated GFPs that cannot emit fluorescence signals can be constructed in a system with an active CRISPR/Cas9 with a guide RNA targeting and repairing the mutated residue in GFP, restores the coding sequence and thus fluorescence. CRISPR/Cas-based genome engineering tools have been widely applied in various plant studies. However, off-target effects of CRISPR/Cas systems or CRISPR-based contaminating gene raise potential biosecurity risks [62]. Hence, a biosensor system to detect CRISPR/Cas systems at an early stage is needed.

In this regard, a plant-based biosensor has been developed using mutated GFP genes for detecting CRISPR/Cas-based genome engineering tools, such as based editors and prime editors [62]. Currently, studies focused on monitoring genome engineering tools are still at an early stage, and the developed biosensors can only monitor specific genome engineering tools. More effort is needed for building universal biosensors monitoring various CRISPR/Cas-based genome engineering invasions.

#### 3.1.2. Validated Components for Extrinsic Direct Plant Biosensors

Extrinsic direct plant biosensors require an additional sensory module to directly interact with analytes. The sensory module can be fused with either a single cpGFP to make a biosensor or two different FPs to build a FRET-based biosensor. One of the most developed and widely applied extrinsic direct plant biosensor is the genetically encoded calcium indicator (GECI). The sensing module for Ca^2+^ is calmodulin (CaM), which recognizes a short polypeptide M13 in target protein [63]. Here, a single GFP GECIs is fused with the calmodulin-M13 to create a single circularly permutated FPs (cpFPs), where the presence of Ca^2+^, calmodulin binds to the M13, inducing conformational changes of FP and inducing its fluorescent properties [30, 64]. In addition, the GCaMP [65] and pericam [66] are two representative GECIs developed based on this strategy. With mutation of several amino acids adjacent to the chromophore, ratiometric-pericam and intensity based-pericam have been developed for monitoring the dynamic change of Ca^2+^ concentration. The cpGFP-based calcium indicator G-GECO has been applied for monitoring the calcium spiking triggered by an arbuscular mycorrhizal fungus in plants [67, 68]. The other widely adopted method for designing GECIs is the FRET-based biosensor. The principle is to flank the Ca^2+^ sensory module, CaM-M13 with two different FPs. Binding of the Ca^2+^ causes calmodulin to bind to M13, thus FRET between the flanking FPs [69]. For example, in yellow Cameleon-2, the CaM-M13 was fused with N-terminal CFP as the FRET donor and C-terminal YFP as the FRET acceptor [69]. FIP-CA indicators are another available design in which only the M13 is linked to two FPs with the engineered CaM fused to the C-terminus of this protein [70].

A similar design strategy has been applied in building plant biosensors for other ions. For instance, high-affinity FRET-based Zn^2+^ biosensors have been developed using two Zinc-binding domain as sensory modules [71]. Two Zn^2+^ binding domains, Atox1 and the fourth domain of ATP7B (WD4), were linked via a flexible linker and flanked by two FPs. This FRET-based biosensor has been applied for measuring the Zn^2+^ concentrations in Arabidopsis root cells [72]. In addition, the cyanobacterial inorganic phosphate, Pi binding protein, has been applied as sensory module flanked by eCFP as FRET donor and different FPs as FRET acceptors for building FRET-based biosensors detecting Pi in plants [73].

Phytohormone receptors can serve as sensory modules for building extrinsic direct phytohormone biosensors. For instance, potential ABA sensory domains (PAS) were selected from members of ABA coreceptor complexes for ABA FRET-based biosensors design [74]. Here, there were two types of designs: a single domain design, in which either a member of PYR/PYL/RCAR family of ABA receptors or a member of the PP2CA subfamily of ABA coreceptor phosphatases alone is inserted between two FPs, and a double domain design, in which the sensory module consists of a PYL/PYR/PCAR fused with a PP2CA via a linker [74]. After screening different ABA biosensor designs, ABACUS1, a FRET-based biosensor built with ABA receptor PYL and coreceptor PP2CAs, is characterized by an affinity of ~80 *μ*M, was selected for ABA [74]. Auxin is another important phytohormone regulating plant development. Auxin biosensors provide effective tools for revealing the dynamics of auxin signaling during many developmental contexts. A novel FRET-based biosensor for auxin has been built using an engineered bacterial tryptophan repressor (TrpR) [12]. To build this biosensor, the selected TrpR variants with high affinity to IAA were flanked by Aquamarine as the FRET donor and mNeonGreen as the FRET acceptor [12].

Any protein conformational change caused by the surrounding environment without direct binding activity can also be monitored by extrinsic direct plant biosensors. Intrinsically disordered regions (IDRs) of a protein are domains that can be sensitive to the physical–chemical properties of its surrounding environment. Utilizing the *Arabidopsis* intrinsically disordered AtLEA4-5 protein, whose confirmational change is induced by water deficit, a FRET biosensor named SED1 was developed for monitoring water-associated stress in living bacteria, yeast, plant, and human cells [75].

### 3.2. Validated Components for Indirect Plant Biosensors

In contrast with direct biosensors, the sensory modules of indirect plant biosensors are involved in various biological processes. Because the readout of indirect plant biosensors is the quantity of reporter present, indirect biosensors are usually intensiometric. A ratiometric readout, though less common, can be obtained with a nonresponsive reporter for normalization. Without relying on the structural change of the FPs, morphological reporters can also be applied for indirect plant biosensors design. In this section, validated representative biological parts of indirect plant-based biosensors will be discussed and are listed in Tables 3–5.

**Table 3:** Selected examples of validated biological parts for transcriptional regulation-based plant biosensors. Promoter sequences for listed “promoter-reporter” systems are provided in supplemental data 3.

Name	Readout	Description	Biosensor target	Tested plant species	References
RD29A::Luc	Intensity	Reporters driven by a native ABA and dehydration-responsive promoter	ABA	*Arabidopsis thaliana*	[76]
DR5::GFP/Luc	Intensity	Reporters driven by a synthetic auxin responsive promoter	Auxin	*A. thaliana*, *Populus tremula*×*alba* 717-1B4	[31, 138]
TCS::GFP	Intensity	Reporters driven by synthetic cytokinin responsive promoters	Cytokinin	*A. thaliana*, *Zea mays*	[139]
FLS2::GUS	Intensity	Reporters driven by salicylic acid responsive promoter	Salicylic acid	*Solanum tuberosum*	[79]
SD::TurboGFP	Intensity	Reporters driven by synthetic water-deficit stress responsive promoters	Water-deficit stress	*A. thaliana*, *P. tremula*×*alba* 717-1B4	[33]
SS16::TurboGFP	Intensity	Reporters driven by synthetic salt stress responsive promoters	Water-deficit stress	*A. thaliana*, *P. tremula*×*alba* 717-1B4	[33]
SCN::RFP	Intensity	Reporters driven by synthetic soybean cyst nematode responsive promoters	Soybean cyst nematode	*Glycine max*	[81]
ER::GUS	Intensity	Reporters driven by synthetic plant pathogen responsive promoters	Plant pathogen	*Nicotiana benthamiana*	[80]
TNT indicator	Intensity	Reporters controlled under a whole synthetic signaling transduction pathway (ssTNT.R3 → Fls-Trg-PhoR → PhoB-VP64 → PlantPho promoter::GUS/De-greening circuits)	TNT	*A. thaliana*	[38]
HACRs	Intensity	Reporters regulated by dCas9 based synthetic TFs (dCas9-degron-TPL repressor gRNAs+pUBQ1::Venus/luciferase)	Phytohormones	*A. thaliana*	[34]

**Table 4:** Selected examples of validated biological parts for posttranslational regulation-based plant biosensors. Coding sequences for listed biosensors are provided in supplemental data 4.

Name	Readout	Description	Biosensor target	Tested plant species	References
DELLA-reporter	Intensity	Proteins containing DELLA domain fused with GFP	Gibberellin	*Arabidopsis thaliana*	[84]
DII-reporter	Intensity	Degron motif of AtIAA28 fused with VENUS	Auxin	*A. thaliana*	[6]
Jas9-reporter	Intensity	Degron motif of JAZ proteins fused with VENUS	Jasmonic acid	*A. thaliana*	[40]
AtSMXL6-reporter	Intensity	SL-responsive degradation protein fused with luciferase	Strigolactone	*A. thaliana*	[86]
DIG1-reporter	Intensity	Conditionally stable plant steroid binding domain	Digoxin and progesterone	*A. thaliana*	[35]
PRG0-reporter	Intensity	Conditionally stable plant steroid ligand binding domain	Digoxin and progesterone	*A. thaliana*	[35]
Fen-reporter	Intensity	Conditionally stable fentanyl binding domain with GFP/Luc	Fentanyl	*A. thaliana*	[87]

**Table 5:** Selected examples of validated biological parts for translocation-based plant biosensors. Coding sequences for listed biosensors are provided in supplemental data 5.

Name	Readout	Description	Biosensor target	Tested plant species	References
BZR1-YFP	Subcellular localization of reporters	BZR1 protein fused with YFP	Brassinosteroid	*Arabidopsis thaliana*	[42]
NLP7-GFP	Subcellular localization of reporters	NLP7 protein fused with GFP	Nitrate	*A. thaliana*	[8]
PIPs indicator	Subcellular localization of reporters	Phospholipids-binding domains fused with FPs	Phosphatidylinositolphosphates (PIPs)	*A. thaliana*	[90]
KLR-MKP1	Subcellular localization of reporters	MKP1 docking domain fused with KLR (MKP1-bNLS-mNeonGreen-T2A-NES-mRuby3-SV40NLS)	Phosphorylation	*A. thaliana*	[96]
KLR-AP2C1	Subcellular localization of reporters	AP2C1 docking domain fused with KLR (AP2C1-bNLS-mNeonGreen-T2A-NES-mRuby3-SV40NLS)	Phosphorylation	*A. thaliana*	[96]
PP7- RNA reporter	Subcellular localization of reporters	An RNA aptamer recruiting PP7 BACTERIOPHAGE COAT PROTEIN fused FPs	Gene expression	*Nicotiana benthamiana*, *A. thaliana*	[49]
MS2- RNA reporter	Subcellular localization of reporters	An RNA aptamer recruiting MS2 COAT PROTEIN fused FPs	Gene expression	*N. benthamiana*, *A. thaliana*	[49]

#### 3.2.1. Validated Components for Transcriptional Regulation-Based Plant Biosensors

The promoter-reporter systems are the simplest design for transcriptional regulation-based biosensors. In plants, promoters control genes expression patterns, and as noted above, promoter-reporter systems have been used for sensing phytohormones. For example, the expression of firefly luciferase driven by the abscisic acid (ABA)-inducible promoter has been developed to sense ABA levels in plants [76, 77]. A similar biosensor for investigating the dynamics of cytokinin using the synthetic promoter, TCS, has also been created [78]. Additionally, salicylic acid (SA) biosensors built with an SA responsive promoter, flagellin sensing 2 promoter, and a synthetic promoter have been utilized for monitoring biotic stress in plants [79].

Currently, novel cis sequences that respond to specific stimuli can be rapidly discovered and identified via bioinformatic tools and high-throughput gene expression profiles [80, 81]. To increase the accuracy of motif prediction, multiple bioinformatic tools should be applied for motif evaluation [81]. Once cis sequences have been identified synthetic responsive promoters can be designed by fusing multiple copies of a motif with a minimal 35S promoter. Both abiotic and biotic responsive synthetic promoters have been developed following this approach [33, 80, 81]. For instance, five synthetic promoters have been developed for building biosensors sensing drought or salt stress [33]. The *de novo* soybean cyst nematode-inducible synthetic promoters have been designed as biosensors monitoring soybean cyst nematode infection [81]. Using this method, pathogen elicitor-responsive synthetic promoters have been made for studying plant-pathogen interactions [80].

The output of promoter-reporter systems may be affected by cross talk from other pathways; the synthetic signal transduction system promises independent regulatory systems for building transcriptional regulation-based plant biosensors. For example, a bacterial periplasmic binding protein (PBP) was redesigned as a trinitrotoluene (TNT) receptor [38]. When TNT binds to this receptor, the confirmational change of the PBP-TNT complex promotes its interaction with a bacterial chemotactic receptor (e.g., Trg). Here, the Trg was fused with a histidine kinase (HK) to produce a chimeric kinase that transfers the TNT binding activity to Phosphorus-dependent gene expression [38]. A synthetic TF PhoB-VP64 and synthetic promoter PlantPho, together with the Trg-HK, makes a complete synthetic signal transduction system. The TNT receptor, together with synthetic signal transduction components and a synthetic reporter degreening gene circuit, were introduced to *Arabidopsis* to build a plant-based biosensor for monitoring TNT [38].

Finally, combining cis elements from yeast with plant minimal promoters has been used to create a set of synthetic promoters with various expression strengths. Together with different TFs, such as Gal4, MADS (MCM1), and GATA (Gat1), an orthogonal regulatory tool box for transcription-based biosensors has been created for plants [36]. Using a phosphate responsive promoter, *AtPht1.1* to drive the synthetic activator, an external phosphate biosensor was made in *Arabidopsis* [36]. In addition, dCas9-based synthetic TFs can be used to provide additional design options for transcription-based biosensors. For example, a synthetic hormone-responsive biosensor has been developed fusing the dCas9-based repressor with phytohormone inducible degrons [34].

#### 3.2.2. Validated Components for Posttranslational Modification-Based Plant Biosensors

Posttranslational modification-based plant biosensors, such as degron-based biosensors, have been successfully design and tested based on ubiquitin-mediated protein degradation [82, 83]. Because proteins under hormone-dependent degradation are often regulators of phytohormone signaling pathways, degron-based biosensors are ideal tools to monitor the change of phytohormones in different plant tissues during various development stages. For example, gibberellin (GA) indicators have been developed by fusing GFP with DELLA proteins to monitor GA levels in hypocotyls during photomorphogenesis [11, 84]. By fusing the FP VENUS with auxin-inducible degron motif DII, a novel auxin biosensor was designed to map auxin distribution with single cell resolution [85]. Similarly, with the jasmonic acid (JA)-responsive degron motif of the JAZ repressors coupled with VENUS, the Jas9-VENUS biosensor was made for analyzing responses to JA in plants with cellular resolution [40]. The readouts of all these three biosensors are intensiometric, whereas the ratiometric version of these biosensors has been made by including another stable reporter. For example, the R2D2, a ratiometric auxin biosensor, has been made by including a mDII-tdTomato reporter for normalization [31]. Another ratiometric degron-based biosensor, StrigoQuant, was engineered by linking AtSMXL6 with a firefly luciferase to quantify strigolactone (SL) activity and specificity [11, 86].

Computational design of ligand binding proteins promises novel components for biosensors sensing small molecules. As mentioned in Section 2, engineered conditionally stable LBDs have also been applied as sensory elements for plant-based biosensors. The computationally designed binding domains, such as DIG0 binding the plant steroid glycoside digoxin, progesterone binder (PRO0), and the potent analgesic fentanyl binder, have been applied in building plant-based biosensors [35, 39, 87]. Fusing these ligand binding domains with degrons and adding a transcriptional activation domain creates responsive synthetic transcriptional factors that can amplify the reporter signal of the biosensors [35].

#### 3.2.3. Validated Components for Translocation-Based Plant Biosensors

Translocation-based biosensors reflect the change of localization of reporters induced by the signals. This type of biosensor provides high resolution at the single cell level. Translocation-based biosensors have been used as tools for monitoring protein localization, phosphorylation activity, and gene expression. As mentioned in Section 2.2.3, the biosensor BZR1-CFP has been used in investigating the spatiotemporal brassinosteroid signaling in *Arabidopsis* [42]. Because the localization of transcription factors NLP7 is regulated by nitrate via nuclear retention mechanism, the localization of NLP7-GFP can be used to monitor the nitrate level in *Arabidopsis* [8]. Similarly, phosphoinositide signaling is mediated by different lipid-binding domains via the direct recruitment of effector proteins to the plasma membrane [88, 89]. A multicolor/multiaffinity marker set has been built by fusing lipid-binding domains with FPs to visualize phosphoinositide dynamics in *Arabidopsis* [90].

Protein phosphorylation plays key roles in signaling networks of cell differentiation, plant development, and stress responses [44]. Kinase translocation reporters (KTRs) have been used to enable spatiotemporal visualization of protein kinase activity in plant cells [91]. Although many KTRs have been developed for mammalian cells, *Caenorhabditis elegans*, and zebrafish [92–95], only two translocation reporters have been designed in plant systems [96]. These two translocation reporters were made by fusing two mitogen-activated protein kinase (MAPKs) docking domains, MKP1 and AP2C1, with the kinase localization reporter (KLR). Both exhibit changes in subcellular localization in response to chitin [96].

It is always a challenge to build a biosensor for detecting the dynamic expression of target genes because most of the output data reflect the change at the protein level using protein-dependent reporters. Currently, PP7 and MS2 RNA labelling technologies have been used for monitoring regions of active transcription in plants [49]. Simpler RNA labeling technologies have been developed with the discovery of fluorescent RNA aptamers. Bai et al. [97] have used a fluorescent RNA aptamer 3WJ-4×Bro, which is a fluorescent tag used for visualizing the transcriptome in real time, to identify transcriptional activity.

## 4. Next-Generation Identification and Design of Biological Components for Engineering Plant-Based Biosensors

Advanced genome engineering technologies (e.g., CRISPR/Cas tools) offer exciting prospects with respect to next-generation plant-based biosensors; however, there are several unique challenges that must be addressed. A challenge of plant-based biosensor design is validation due to tedious tissue- or cell-level assays. Advancements in protein-engineering technologies are largely hampered by the lack of high-throughput screening systems in plants. To solve this problem, single cell transient assay systems using FACS could be applied for high-throughput screening in plants. Apart from these technical challenges, plant biosystems have inherently complex circuitry logic, spanning and intersecting multiple layers of biochemical, molecular, and cellular processes. Engineering across these multiple layers remains a grand challenge [98]. Additional biological components, involved in alternate levels of gene regulation, are still needed for addressing this challenge. In this section, principles and strategies of designing biological parts for engineering plant-based biosensors will be discussed.

### 4.1. Principles for the Design of Sensory Elements for Plant Biosensors

To design extrinsic direct biosensors and indirect biosensors, domains, motifs, promoters, etc. can be used as sensory modules for targeted or specific analytes. In the first approach, conformational changes of the sensory module is caused by binding with the signal molecules. In the second approach, the sensory module is used to regulate the transcription level of reporters by binding with the analyte. Native and synthetic responsive promoters are sensory modules with this feature. In the third approach, the sensory module is used to change the translational level of reporters and thus give a readout when sensing the signal molecules. In the fourth approach, binding with the analyte is used to change localization of the biosensor and thus the movement of the biosensor is used as a readout.

In addition to the four features, analyte binding specificity and affinity are two additional key factors that should be considered when designing the sensory module in biosensors. These two factors will influence the sensitivity of biosensors. Computational design of ligand-binding domains has also been applied to engineer LBDs with higher specificity and affinity [35, 39, 87].

### 4.2. Principles for Choosing Reporters for Plant Biosensors

There are two types of reporter modules, optical reporters (FPs and Luc) and morphological reporters (e.g., color markers and herbicide/antibiotic resistant marker). Optical reporters can be applied for monitoring signal changes at the cellular level, whereas the morphological reporters are usually used to reflect signal changes at the whole-plant level. The choice of reporters for direct biosensors is limited to optical reporters. For indirect biosensors, other than optical reporters, morphological reporters can be applied. Emerging examples for application of morphological reporters are the synthetic RUBY markers that have been being widely utilized for transgenic plant selection [99].

Identifying the ideal reporter is important for plant biosensor design. Key to this identification is an easily detectable signal. Fluorescent proteins are being implemented for biosensor design because their fluorescence signals can be reliably detected and imaged. A new reporter RUBY that converts tyrosine to vividly red betalain can be detected without the need of special equipment or chemical treatment [99]. Another potential reporter for biosensor is eYGFPuv, which has been successfully used as a reporter for plant transformation [100, 101]. Both RUBY and eYGFPuv could be used as suitable reporters for building transcriptional- or translational-based biosensors. Additionally, reporters fused to a sensory module need themself to be insensitive to the analyte. Likewise, the reporters should be expressed efficiently resulting in no toxicity to the chosen system [100]. Another important factor for choosing the reporter is the maturation time and half-life. This factor is especially important for building degron-based biosensors. For example, the VENUS fast maturing yellow fluorescent protein (YFP) has been fused with DII domain to overcome the technical limitation of GFP maturation time, where GFP maturation time is often longer than Aux/IAA half-lives [6]. Alternatively, many biosensors need to monitor signals in real time. The accumulation of proteins may not allow the biosensor to reflect the real-time change of the signal. In this case, a reporter with a short half-life should be considered. Luciferase reaction can emit bioluminescence within a short time frame, and therefore a luciferase reporter is also a good choice for real-time signal monitoring.

### 4.3. Identification of Natural Biological Components for Biosensor Engineering in Plants

Plant system biology has provided a large amount of biological parts for biosensor design from its detailed characterization of molecular components in plant systems [102]. Many sensory modules are being discovered through basic plant biology studies. The knowledge of protein-protein interactions and upstream regulators of signaling pathways is essential for choosing the right parts for designing biosensors. Currently, the rapid accumulation of omics data is accelerating progress towards gene, promoter, and motif discovery [103].

Transgenic overexpression lines and knock-out mutants are used to validate predicted parts. However, traditional plant tissue culture is time consuming and labor intensive, creating a bottleneck for parts mining. Recently, progress has been made in plant transformation using developmental regulators. For example, overexpression of developmental regulators, such as *Baby Boom*, *Wuschel*, GROWTH-REGULATING FACTOR 4 (GRF4), and its cofactor GRF-INTERACTING FACTOR 1 (GIF1), have dramatically increased the efficiency and speed of regeneration in different plant species [104, 105]. In addition, developmental regulators have been used together with gene editing reagents to generate gene-edited plants through *de novo* meristem induction, thereby, bypassing the tissue culture process [106].

### 4.4. Modification of Natural Biological Components for Biosensor Engineering in Plants

Naturally occurring biological components are involved in complex networks and usually involve crosstalk with different signaling pathways. The lack of understanding of the mechanisms involved in signaling pathway crosstalk is limiting for biosensor development. An alternate route to the modification of natural biological components may be achieved via protein engineering. Directed evolution has been a successful method for protein engineering. This approach has been used to generate a library of variants followed by screening to identify desirable designs [107]. Computational modeling offers another approach for protein engineering. For instance, using computational modeling, metal binding sites have been engineered into green fluorescence protein for sensing Cu^2+^, Ni^2+^, or Co^2+^ [108]. The features of naturally occurring binding sites should be considered when developing a computational method for engineering ligand-binding proteins [39]. Such that, the binding of the ligands should be energetically favorable. Secondly, the overall shape of the binding site should be complementary to the ligand. Thirdly, the structure of the unbound protein should be stable. Based on these principles, a robust computational method for the design of high affinity and specificity ligand-binding-proteins has been developed [39]. Finally, machine-learning-guided directed evolution has been applied in protein engineering to enable the optimization of protein functions in a data driven manner [107]. Longstanding campaigns around protein structure design have successfully been used to design new biosensors [109].

### 4.5. Design of New-to-Nature Components for Engineering Biosensors in Plants

The engineering of synthetic plant-based biosensors requires components that allow for sensing, signal processing, and output [98]. The synthetic plant-based biosensors consist of orthogonal components that function independent from the plant endogenous signaling pathway or network. A synthetic promoter can be designed by combining a core promoter region with signal-responsive motifs serving as the sensing module for a transcriptional-based biosensor [98, 110]. The most widely used core promoter is the minimal CaMV 35S promoter. Currently, three databases, PLACE, PlantCARE, and TRANSFAC, are available for the selection of cis-regulatory elements with known functions [110].

AI-based frameworks are being applied for promoter analysis and prediction in plants [111, 112]. The TSSP-TCM was the first tool to predict and identify plant promoters using the support vector machine (SVM) algorithm [113]. Currently, many computational tools of plant promoter prediction have been developed [114–116]. The PromPredict program using DNA degree of stability features of promoter regions showed accurate promoter prediction in *Arabidopsis* and rice genomes [117]. AI-based frameworks for *de novo* promoter design and prediction have also been developed in *E. coli* [118, 119]. These AI-based frameworks lay the foundation for the future promoter design work in plants. The conserved phytohormone-responsive and stress-inducible cis elements have been identified via overlapping results of various bioinformatical tools [33, 120–122].

Another useful class of biological components is based on synthetic transcription factors (TFs). Utilizing DNA-binding domains and activation domains from different organisms, synthetic TFs have been constructed. For instance, the transcription activator-like effectors (TALEs), dead Cas9 fused with VP64, and others, have been used to build synthetic TFs in plants [110, 123].

In addition to synthetic sensory modules, synthetic reporters are also important for engineering plant-based biosensors. Although the FPs and luciferase can generate facile readout signals, the requirement of fluorescence microscopes and expensive substrate limited the use of these reporters. Currently, several synthetic reporters that can overcome these limitations have been developed for plant system. For instance, the synthetic RUBY reporter, including all the enzymes for betalain biosynthesis, has been generated to visualize transgenic events [99]. A fungal bioluminescence pathway (FBP) can create luminescence *in planta* without additional substrate addition [124].

### 4.6. The Challenges Specific to Constructing a Multicellular Biosensor to Monitor the Environment

One of the advantages of using plants to build sensors for environmental monitoring is their ability to change morphology. Plant tissues and cells deeply penetrate the soil and extend up into the air, enabling them to sample the environment broadly and continuously. While this morpho-physiology is beneficial for sampling, it creates unique challenges for the implementation of GEPBs. For example, if a signal is sensed in one or a few cells in the plant, only those cells would report it. Thus, unless every cell was monitored continuously, which is impractical and infeasible, the detected signal would be invisible. Additionally, if the signal was perceived in cells that were hidden from view, such as roots in soil, the signal would not be visible unless the plant was excavated. These challenges indicate that biosensors need to be created with systems capable of amplifying reporter signals and transmitting them throughout the plant.

Signal amplification is essential to convert signal perception in a subset of cells in the plant to a response that is easily measured. There are several synthetic signaling systems that have been developed, such as transcriptional feedback loops [125] and engineered kinase cascades [126, 127], that can be used to engineer systemic reporter signal amplification. However, such systems would need to be calibrated to prevent auto-activation or a loss of correlation between the strength of a signal and the reporter output. Once locally amplified, this signal would also need to be transmitted to regions of the plant that were accessible to measurement for the plant-based biosensor to be useful. Engineering the mobility of reporter signal through the plant could be implemented at either the RNA or protein level. At the RNA level, fusion of tRNA-like sequences to messenger RNAs has been demonstrated to confer systemic mobility to them, enabling transport through the phloem [128, 129]. This strategy has been utilized by groups to both confer mobility to reporters across graft junctions and to enhance the mobility of viral vectors [128, 129]. At the protein level, reporter mobility could be engineered by cloning domains from the movement proteins of plant viruses, which have evolved to enable such transport [130]. Both these strategies are generic and could be broadly applied to many different reporters.

## 5. Conclusion

The goal of engineering for plant-based biosensors is to design and build biosensors with high specificity and sensitivity. It would be ideal to engineer plant-based biosensors to reflect real-time dynamics of analytes without the need for complicated equipment to detect the signal.

In this review, we outline a general framework for engineering different type of biosensors. We highlight representative biological components (e.g., FPs, natural sensory modules, synthetic reporters, and synthetic sensory modules) that have been successfully applied in plant-based biosensors design, and we conclude with a look to the future discussing synthetic sensory modules.

The items listed in Tables 1–5 serve as a guide for generating a more comprehensive catalog of biological components for GEPBs. We propose the following strategies for identification and curation of more biological components for plant-based biosensor engineering:

(1) Selecting the promoters of responsive genes to specific analytes, TFs responsive to specific signals, or proteins that interact with analytes from the literature to develop plant-based biosensors for specific analytes
(2) Designing new synthetic biological components for plant-based biosensor engineering with *de novo* protein design
(3) Applying new natural or synthetic reporters with facile readout properties for plant-based biosensor engineering

Currently, it is still a challenge to identify biological components in order to build reliable plant-based biosensors for various analytes. This is, in part, due to the fact that molecular events in plants are complicated and the function of many biological components still need further characterization. Second, large genomic databases or resources are lacking for many plant species, which makes it difficult to apply AI-based modeling for designing biological components for plant-based biosensor engineering. Third, high-throughput screening systems are also lacking in plants for validating new biological components for plant-based biosensor engineering. All these challenges represent substantial opportunities for future plant synthetic biology research. Additional efforts are also needed in plant biotechnology, including improved CRISPR-based genome engineering, rapid transformation systems, and high-throughput phenotyping systems, which would accelerate Design-Build-Test-Learn cycles for plant synthetic biology.
