# Supplementary material for: Biological and Molecular Components for Genetically Engineering Biosensors in Plants
Source: Biodes Res. 2022 Nov 9;2022:9863496. doi: 10.34133/2022/9863496 (PMC10521658; doi:10.34133/2022/9863496)
Supplement: Supplementary Materials — Coding sequences for listed biosensors are provided in supplemental data 1-supplemental data 5. [file 9863496.f1.zip › Supplemental data 1 Sequences for biosensors in Table 1.pdf]

>pHusion GFP cds in green, RFP cds in purple, linker in grey. (Note: CDS sequences are codon optimized for *Arabidopsis thaliana* from protein sequences.)
ATGGCTTCCTCCGAGGACGTTATCAAAGAGTTCATGCGTTTCAAAGTTCGTATGGAAGGTTCCGTTAAC
GGTCACGAGTTCGAAATCGAAGGTGAAGGTGAAGGTCGTCCGTACGAAGGTACCCAGACCGCTAAAC
TGAAAGTTACCAAAGGTGGTCCGCTGCCGTTCGCTTGGGACATCCTGTCCCCGCAGTTCCAGTACGGTT
CCAAAGCTTACGTTAAACACCCGGCTGACATCCCGGACTACCTGAAACTGTCCTTCCCGGAAGGTTTC
AAATGGGAACGTGTTATGAACTTCGAGGACGGTGGTGTTGTTACCGTTACCCAGGACTCCTCCCTGCA
AGACGGTGAGTTCATCTACAAAGTTAAACTGCGTGGTACCAACTTCCCGTCCGACGGTCCGGTTATGC
AGAAAAAAACCATGGGTTGGGAAGCTTCCACCGAACGTATGTACCCGGAGGACGGTGCTCTGAAAGG
TGAAATCAAAATGCGTCTGAAACTGAAAGACGGTGGTCACTACGACGCTGAAGTTAAAACCACCTAC
ATGGCTAAAAAACCGGTTCAGCTGCCGGGTGCTTACAAAACCGACATCAAACTGGACATCACCTCCCA
CAACGAGGACTACACCATCGTTGAACAGTACGAACGTGCTGAAGGTCGTCACTCCACCGGTGCTTAAG
CAGTAAACGCATCAATGGTGAGCAAGGGCGAGGAGCTGTTCACCGGGGTGGTGCCCATCCTGGTCGA
GCTGGACGGCGACGTAAACGGCCACAAGTTCAGCGTGTCTGGCGAGGGCGAGGGCGATGCCACCTAC
GGCAAGCTGACCCTGAAGTTCATCTGCACCACCGGCAAGCTGCCCGTGCCCTGGCCCACCCTCGTGAC
CACCTTCACCTACGGCGTGCAGTGCTTCAGCCGCTACCCCGACCACATGAAGCAGCACGACTTCTTCA
AGTCCGCCATGCCCGAAGGCTACGTCCAGGAGCGCACCATCTTCTTCAAGGACGACGGCAACTACAAG
ACCCGCGCCGAGGTGAAGTTCGAGGGCGACACCCTGGTGAACCGCATCGAGCTGAAGGGCATCGACT
TCAAGGAGGACGGCAACATCCTGGGGCACAAGCTGGAGTACAACTACAACAGCCACAACGTCTATAT
CATGGCCGACAAGCAGAAGAACGGCATCAAGGCGAACTTCAAGATCCGCCACAACATCGAGGACGGC
AGCGTGCAGCTCGCCGACCACTACCAGCAGAACACCCCCATCGGCGACGGCCCCGTGCTGCTGCCCGA
CAACCACTACCTGAGCACCCAGTCCGCCCTGAGCAAAGACCCCAACGAGAAGCGCGATCACATGGTC
CTGCTGGAGTTCGTGACCGCCGCCGGGATCACTCTCGGCATGGACGAGCTGTACAAGTAA

>PE-pHluorin cds (Note: CDS sequences are codon optimized for *Arabidopsis thaliana* from protein sequences.)
GGATCCAAGGAGATATAACAATGAGTAAAGGAGAAGAACTTTTCACTGGAGTTGTCCCAATTCTTGTT
GAATTAGATGGTGATGTTAATGGGCACAAATTTTCTGTCAGTGGAGAGGGTGAAGGTGATGCAACATA
CGGAAAACTTACCCTTAAATTTATTTGCACTACTGGAAAACTACCTGTTCCATGGCCAACACTTGTCAC
TACTTTCTCTTATGGTGTTCAATGCTTTTCAAGATACCCAGATCATATGAAGCGGCACGACTTCTTCAA
GAGCGCCATGCCTGAGGGATACGTGCAGGAGAGGACCATCTCTTTCAAGGACGACGGGAACTACAAG
ACACGTGCTGAAGTCAAGTTTGAGGGAGACACCCTCGTCAACAGGATCGAGCTTAAGGGAATCGATTT
CAAGGAGGACGGAAACATCCTCGGCCACAAGTTGGAATACAACTACAACTCCCACAACGTATACATC
ACGGCAGACAAACAAAAGAATGGAATCAAAGCTAACTTCAAAATTAGACACAACATTGAAGATGGAA
GCGTTCAACTAGCAGACCATTATCAACAAAATACTCCAATTGGCGATGGCCCTGTCCTTTTACCAGAC
AACCATTACCTGTCCACACAATCTGCCCTTTCGAAAGATCCCAACGAAAAGAGAGACCACATGGTCCT
TCTTGAGTTTGTAACAGCTGCTGGGATTACACATGGCATGGATGAACTATACAAATAAGAGCTCATGA
GTAAAGGAGAAGAACTTTTCACTGGAGTTGTCCCAATTCTTGTTGAATTAGATGGTGATGTTAATGGG
CACAAATTTTCTGTCAGTGGAGAGGGTGAAGGTGATGCAACATACGGAAAACTTACCCTTAAATTTAT
TTGCACTACTGGAAAACTACCTGTTCCATGGCCAACACTTGTCACTACTTTCTCTTATGGTGTTCAATG
CTTTTCAAGATACCCAGATCATATGAAACGGCATGACTTTTTCAAGAGTGCCATGCCCGAAGGTTATGT
ACAGGAAAGAACTATATTTTTCAAAGATGACGGGAACTACAAGACACGTGCTGAAGTCAAGTTTGAA
GGTGATACCCTTGTTAATAGAATCGAGTTAAAAGGTATTGATTTTAAAGAAGATGGAAACATTCTTGG
ACACAAATTGGAATACAACTATAACGATCACCAGGTGTACATCATGGCAGACAAACAAAAGAATGGA
ATCAAAGCTAACTTCAAAATTAGACACAACATTGAAGATGGAGGCGTTCAACTAGCAGACCATTATCA
ACAAAATACTCCAATTGGCGATGGGCCCGTCCTTTTACCAGACAACCATTACCTGTTTACAACTTCTAC
TCTTTCGAAAGATCCCAACGAAAAGAGAGACCACATGGTCCTTCTTGAGTTTGTAACAGCTGCTGGGA
TTACACATGGCATGGATGAACTATACAAATAA

>PR-pHluorin cds (Note: CDS sequences are codon optimized for *Arabidopsis thaliana* from protein sequences.)
GGATCCAAGGAGATATAACAATGAGTAAAGGAGAAGAACTTTTCACTGGAGTTGTCCCAATTCTTGTT
GAATTAGATGGTGATGTTAATGGGCACAAATTTTCTGTCAGTGGAGAGGGTGAAGGTGATGCAACATA
CGGAAAACTTACCCTTAAATTTATTTGCACTACTGGAAAACTACCTGTTCCATGGCCAACACTTGTCAC
TACTTTCTCTTATGGTGTTCAATGCTTTTCAAGATACCCAGATCATATGAAGCGGCACGACTTCTTCAA
GAGCGCCATGCCTGAGGGATACGTGCAGGAGAGGACCATCTCTTTCAAGGACGACGGGAACTACAAG
ACACGTGCTGAAGTCAAGTTTGAGGGAGACACCCTCGTCAACAGGATCGAGCTTAAGGGAATCGATTT
CAAGGAGGACGGAAACATCCTCGGCCACAAGTTGGAATACAACTACAACTCCCACAACGTATACATC

ACGGCAGACAAACAAAAGAATGGAATCAAAGCTAACTTCAAAATTAGACACAACATTGAAGATGGAA
GCGTTCAACTAGCAGACCATTATCAACAAAATACTCCAATTGGCGATGGCCCTGTCCTTTTACCAGAC
AACCATTACCTGTCCACACAATCTGCCCTTTCGAAAGATCCCAACGAAAAGAGAGACCACATGGTCCT
TCTTGAGTTTGTAACAGCTGCTGGGATTACACATGGCATGGATGAACTATACAAATAAGAGCTCATGA
GTAAAGGAGAAGAACTTTTCACTGGAGTTGTCCCAATTCTTGTTGAATTAGATGGTGATGTTAATGGG
CACAAATTTTCTGTCAGTGGAGAGGGTGAAGGTGATGCAACATACGGAAAACTTACCCTTAAATTTAT
TTGCACTACTGGAAAACTACCTGTTCCATGGCCAACACTTGTCACTACTTTCTCTTATGGTGTTCAATG
CTTTTCAAGATACCCAGATCATATGAAACGGCATGACTTTTTCAAGAGTGCCATGCCCGAAGGTTATGT
ACAGGAAAGAACTATATTTTTCAAAGATGACGGGAACTACAAGACACGTGCTGAAGTCAAGTTTGAA
GGTGATACCCTTGTTAATAGAATCGAGTTAAAAGGTATTGATTTTAAAGATGATGGAAACATTCTTGG
ACACAAATTGGAATACAACTATAACGAGCACTTGGTGTACATCATGGCAGACAAACAAAAGAATGGT
ACCAAAGCTATCTTTCAAGTTCACCACAACATTGAAGATGGAGGCGTTCAACTAGCAGACCATTATCA
ACAAAATACTCCAATTGGCGATGGCCCTGTCCTTTTACCAGACAACCATTACCTGCACACACAATCTGC
CCTTTCGAAAGATCCCAACGAAAAGAGAGACCACATGGTCCTTCTTGAGTTTGTAACAGCTGCTGGGA
TTACACATGGCATGGATGAACTATACAAATAA

>Pt-GFP cds (Note: CDS sequences are codon optimized for *Arabidopsis thaliana* from protein sequences.)
CTGGCCTCCACACTTTAGACAAAATGAACCGCAACGTATTAAAGAACACTGGACTGAAAGAGATTATG
TCGGCAAAAGCTAGCGTTGAAGGAATCGTGAACAATCACGTTTTTTCCATGGAAGGATTTGGAAAAGG
CAATGTATTATTTGGAAACCAATTGATGCAAATCCGGGTTACAAAGGGAGGTCCGTTGCCATTCGCTTT
CGATATTGTTTCCATAGCTTTCCAATACGGGAATCGCACTTTCACGAAATACCCAGACGACATTGCGG
ACTACTTTGTTCAATCATTCCCGGCTGGATTTTTCTACGAAAGAAATCTACGCTTTGAAGATGGCGCCA
TTGTTGACATTCGTTCAGATATAAGTTTAGAAGATGATAAGTTCCACTACAAAGTGGAGTATAGAGGC
AACGGTTTCCCTAGTAACGGACCCGTGATGCAAAAAGCCATCCTCGGCATGGAGCCATCGTTTGAGGT
GGTCTACATGAACAGCGGCGTTCTGGTGGGCGAAGTAGATCTCGTTTACAAACTCGAGTCAGGGAACT
ATTACTCGTGCCACATGAAAACGTTTTACAGATCCAAAGGTGGAGTGAAAGAATTCCCGGAATATCAC
TTTATCCATCATCGTCTGGAGAAAACCTACGTGGAAGAAGGAAGCTTCGTGGAACAACACGAGACGG
CCATTGCACAACTGACCACAATTGGAAAACCTCTGGGCTCCCTTCATGAATGGGTGTAGAAAATGACC
AATATACTGGGGAAACCGATAACCGTTTGGAAGCTTGTGTATACAAATTATTTGGGGTCATTTTGTAAT
GTGTATGTGTGTTGTATGATCAATAGACGTCGTCATTCATAGCTTGAATCCTTCAGCAAAAGAAACCTC
GAAGCATATTGAAACCTCGAAGCATATTGAAACCTCGACGGAGAGCGTAAAGAGACCGCACAAATTA
ACGCGTTTCAACCAGCAGTTGGAATCTTTAAACCGATCAAAACTATTAATATAAATATATATACCCTGT
ATAACTTATATATATCTATATAGTTTGATATTGATTAAATCTGTTCTTGATCAAAAAAAAAAAAAAAAA
AA

>Acidin2 RFP cds in purple, BFP cds in blue, linker in grey. (Note: CDS sequences are codon optimized for *Arabidopsis thaliana* from protein sequences.)
TCTAGAGGGATACGCACGAGTTTCAACTCAAGGAGGATCTAAATGAATTCAGATATGGTTTCAAAAGG
GGAGGAGGATAATATGGCGATTATCAAGGAATTTATGCGGTTTAAAGTGCATATGGAAGGGTCGGTCA
ATGGTCATGAATTTGAGATTGAAGGTGAGGGCGAAGGGCGTCCGTATGAAGGTACGCAGACGGCGAA
GTTAAAAGTCACTAAGGGAGGTCCGCTGCCATTTGCTTGGGATATTTTGAGTCCACAGTTCATGTATGG
ATCGAAAGCTTACGTAAAGCACCCTGCCGATATTCCGGATTATTTGAAGTTAAGTTTTCCAGAAGGATT
TAAGTGGGAACGTGTTATGAACTTTGAAGACGGTGGCGTTGTTACAGTAACTCAAGATTCTTCGTTGC
AAGATGGTGAATTTATTTACAAGGTTAAGTTGCGAGGTACGAATTTTCCGTCAGATGGACCAGTAATG
CAAAAGAAAACTATGGGTTGGGAAGCGAGCAGTGAACGCATGTATCCAGAAGATGGCGCTTTGAAAG
GTGAAATTAAACAGCGGTTGAAATTAAAAGATGGTGGTCACTATGATGCGGAAGTCAAAACGACTTAT
AAAGCTAAGAAACCAGTTCAACTGCCAGGCGCATATAACGTCAATATTAAGCTGGATATCACGAGTCA
TAATGAAGACTATACGATCGTCGAACAGTATGAACGAGCAGAGGGACGACACTCAACAGGTGGCATG
GACGAATTATATAAATGAGCTCGGCAGCATGGTGTCTAAGGGCGAAGAGCTGATTAAGGAGAACATG
CACATGAAGCTGTACATGGAGGGCACCGTGGACAACCATCACTTCAAGTGCACATCCGAGGGCGAAG
GCAAGCCCTACGAGGGCACCCAGACCATGAGAATCAAGGTGGTCGAGGGCGGCCCTCTCCCCTTCGCC
TTCGACATCCTGGCTACTAGCTTCCTCTACGGCAGCAAGACCTTCATCAACCACACCCAGGGCATCCCC
GACTTCTTCAAGCAGTCCTTCCCTGAGGGCTTCACATGGGAGAGAGTCACCACATACGAAGACGGGGG
CGTGCTGACCGCTACCCAGGACACCAGCCTCCAGGACGGCTGCCTCATCTACAACGTCAAGATCAGAG
GGGTGAACTTCACATCCAACGGCCCTGTGATGCAGAAGAAAACACTCGGCTGGGAGGCCTTCACCGA

GACGCTGTACCCCGCTGACGGCGGCCTGGAAGGCAGAAACGACATGGCCCTGAAGCTCGTGGGCGGG
AGCCATCTGATCGCAAACGCCAAGACCACATATAGATCCAAGAAACCCGCTAAGAACCTCAAGATGC
CTGGCGTCTACTATGTGGACTACAGACTGGAAAGAATCAAGGAGGCCAACAACGAGACCTACGTCGA
GCAGCACGAGGTGGCAGTGGCCAGATACTGCGACCTCCCTAGCAAACTGGGGCACAAGCTTAAT

> Acidin3 RFP cds in purple, Gamillus cds in blue, linker in grey. (Note: CDS sequences are codon optimized for *Arabidopsis thaliana* from protein sequences.)
TCTAGAGGGATACGCACGAGTTTCAACTCAAGGAGGATCTAAATGAATTCAGATATGGTTTCAAAAGG
GGAGGAGGATAATATGGCGATTATCAAGGAATTTATGCGGTTTAAAGTGCATATGGAAGGGTCGGTCA
ATGGTCATGAATTTGAGATTGAAGGTGAGGGCGAAGGGCGTCCGTATGAAGGTACGCAGACGGCGAA
GTTAAAAGTCACTAAGGGAGGTCCGCTGCCATTTGCTTGGGATATTTTGAGTCCACAGTTCATGTATGG
ATCGAAAGCTTACGTAAAGCACCCTGCCGATATTCCGGATTATTTGAAGTTAAGTTTTCCAGAAGGATT
TAAGTGGGAACGTGTTATGAACTTTGAAGACGGTGGCGTTGTTACAGTAACTCAAGATTCTTCGTTGC
AAGATGGTGAATTTATTTACAAGGTTAAGTTGCGAGGTACGAATTTTCCGTCAGATGGACCAGTAATG
CAAAAGAAAACTATGGGTTGGGAAGCGAGCAGTGAACGCATGTATCCAGAAGATGGCGCTTTGAAAG
GTGAAATTAAACAGCGGTTGAAATTAAAAGATGGTGGTCACTATGATGCGGAAGTCAAAACGACTTAT
AAAGCTAAGAAACCAGTTCAACTGCCAGGCGCATATAACGTCAATATTAAGCTGGATATCACGAGTCA
TAATGAAGACTATACGATCGTCGAACAGTATGAACGAGCAGAGGGACGACACTCAACAGGTGGCATG
GACGAATTATATAAATGAGCTCGCAGTAAACGCAATGGTGAGCAAGGGCGAGGAGGCATCTGGCAGA
GCCCTGTTCCAGTACCCCATGACCAGCAAGATCGAGCTGAACGGCGAGATCAACGGCAAGAAATTCA
AGGTGGCCGGCGAGGGCTTCACCCCCAGCAGCGGCAGATTCAACATGCACGCCTACTGCACCACCGGC
GACCTGCCTATGAGCTGGGTCGTGATTGCCAGCCCCCTCCAGTACGGCTTCCACATGTTCGCCCACTAC
CCCGAGGACATCACACACTTTTTCCAGGAATGCTTCCCCGGCAGCTACACCCTGGACCGGACCCTGAG
AATGGAAGGCGACGGCACCCTGACCACCCACCACGAGTACAGCCTGGAGGACGGCTGCGTGACCTCC
AAGACCACCCTGAATGCCAGCGGCTTCGACCCTAAGGGCGCCACCATGACCAAGAGCTTCGTGAAAC
AACTGCCTAACGAGGTGAAGATCACCCCCCACGGCCCCAACGGCATCAGACTGACCAGCACCGTGCTG
TACCTGAAGGAGGATGGCACCATCCAGATCGGCACCCAGGACTGCATCGTGACCCCTGTGGGCGGAA
GGAAAGTGACCCAGCCCAAGGCCCACTTCCTGCACACCCAGATCATCCAGAAGAAGGACCCCAACGA
CACCCGGGACCACATCGTGCAGACAGAACTGGCCGTGGCCGGCAATCTGTGGCACGGCATGGACGAG
CTGTACAAGTAA

> Acidin4 RFP cds in purple, SYFP2 cds in yellow, linker in grey. (Note: CDS sequences are codon optimized for *Arabidopsis thaliana* from protein sequences.)
TCTAGAGGGATACGCACGAGTTTCAACTCAAGGAGGATCTAAATGAATTCAGATATGGTTTCAAAAGG
GGAGGAGGATAATATGGCGATTATCAAGGAATTTATGCGGTTTAAAGTGCATATGGAAGGGTCGGTCA
ATGGTCATGAATTTGAGATTGAAGGTGAGGGCGAAGGGCGTCCGTATGAAGGTACGCAGACGGCGAA
GTTAAAAGTCACTAAGGGAGGTCCGCTGCCATTTGCTTGGGATATTTTGAGTCCACAGTTCATGTATGG
ATCGAAAGCTTACGTAAAGCACCCTGCCGATATTCCGGATTATTTGAAGTTAAGTTTTCCAGAAGGATT
TAAGTGGGAACGTGTTATGAACTTTGAAGACGGTGGCGTTGTTACAGTAACTCAAGATTCTTCGTTGC
AAGATGGTGAATTTATTTACAAGGTTAAGTTGCGAGGTACGAATTTTCCGTCAGATGGACCAGTAATG
CAAAAGAAAACTATGGGTTGGGAAGCGAGCAGTGAACGCATGTATCCAGAAGATGGCGCTTTGAAAG
GTGAAATTAAACAGCGGTTGAAATTAAAAGATGGTGGTCACTATGATGCGGAAGTCAAAACGACTTAT
AAAGCTAAGAAACCAGTTCAACTGCCAGGCGCATATAACGTCAATATTAAGCTGGATATCACGAGTCA
TAATGAAGACTATACGATCGTCGAACAGTATGAACGAGCAGAGGGACGACACTCAACAGGTGGCATG
GACGAATTATATAAATGAGCTCGCAGTAAACGCAATGGTGAGCAAGGGCGAGGAGCTGTTCACCGGG
GTGGTGCCCATCCTGGTCGAGCTGGACGGCGACGTAAACGGCCACAAGTTCAGCGTGTCCGGCGAGG
GCGAGGGCGATGCCACCTACGGCAAGCTGACCCTGAAGCTGATCTGCACCACCGGCAAGCTGCCCGTG
CCCTGGCCCACCCTCGTGACCACCCTGGGCTACGGCGTGCAGTGCTTCGCCCGCTACCCCGACCACAT
GAAGCAGCACGACTTCTTCAAGTCCGCCATGCCCGAAGGCTACGTCCAGGAGCGCACCATCTTCTTCA
AGGACGACGGCAACTACAAGACCCGCGCCGAGGTGAAGTTCGAGGGCGACACCCTGGTGAACCGCAT
CGAGCTGAAGGGCATCGACTTCAAGGAGGACGGCAACATCCTGGGGCACAAGCTGGAGTACAACTAC
AACAGCCACAACGTCTATATCACCGCCGACAAGCAGAAGAACGGCATCAAGGCCAACTTCAAGATCC
GCCACAACATCGAGGACGGCGGCGTGCAGCTCGCCGACCACTACCAGCAGAACACCCCCATCGGCGA
CGGCCCCGTGCTGCTGCCCGACAACCACTACCTGAGCTACCAGTCCAAGCTGAGCAAAGACCCCAACG

AGAAGCGCGATCACATGGTCCTGCTGGAGTTCGTGACCGCCGCCGGGATCACTCTCGGCATGGACGAG
CTGTACAAGTAA

> Clomeleon CFP cds in green, YFP cds in purple, linker in grey. (Note: CDS sequences are codon optimized for *Arabidopsis thaliana* from protein sequences.)
ATGTCCAAGGGAGAAGAGCTTTTTACGGGGGTTGTACCCATATTAGTCGAGCTAGATGGTGACGTGAA
TGGTCACAAATTCTCTGTTTCAGGTGAGGGAGAGGGCGACGCAACATATGGAAACTCACGTTAAAAT
TCATTTGTACTACAGGCAAACTTCCAGTTCCGTGGCCTACATTAGTAACTACTTTCAGTTGGGGCGTTC
AGTGCTTTAGTAGGTACCCGGACCATATGAAGCAACACGACTTCTTCAAGTCCGCGATGCCGGAAGGA
TATGTTCAGGAGCGTACTATCTTTTTCAAAGACGATGGAAATTACAAGACACGAGCAGAAGTAAAATT
TGAGGGCGATACTCTCGTAAATAGAATAGAATTAAAAGGTATAGACTTCAAGGAAGATGGAATATTT
TAGGGCACAAACTCGAATACAATTATAATAGTCACAACGTATATATTATGGCAGATAAGCAAAAGAA
CGGTATTAAGGTGAACTTTAAGATCAGACACAACATCGAAGATGGGTCAGTACAATTAGCAGATCATT
ACCAGCAAAACACACCTATTGGTGATGGCCCAGTTTTACTACCTGATAACCATTACCTGTCAACTCAA
AGTGCGCTATCAAAGGATCCTAATGAGAAGCGAGATCACATGGTGCTACTTGAATTCGTCACGGCGGC
AGGCATAACACACGGTATGGATGAACTTTACAAAGAAAACCTATATTTCCAGATGTCCAAGGGTGAAG
AGCTCTTCACGGGTGTGGTTCCCATACTAGTCGAGCTTGACGGGGATGTCAATGGGCATAAATTTAGC
GTTTCAGGCGAGGGTGAAGGAGATGCGACATATGGTAAACTCACATTAAAACTGCTTTGCACCACCGG
GAAACTTCCGGTTCCGTGGCCAACTTTAGTCACAACCTTCGGGTACGGCCTCCAGTGTTTCGCCCGTTA
TCCCGACCATATGAAGAGACACGATTTTTTCAAAAGCGCAATGCCAGAAGGCTATGTTCAGGAGAGGA
CGATATTTTTCAAGGACGACGGAAACTATAAGACGCGTGCAGAGGTCAAGTTTGAAGGAGATACGCTT
GTCAATCGAATTGAACTCAAGGGGATAGATTTTAAGGAAGACGGTAACATATTAGGACACAAGCTAG
AGTATAATTACAATTCACACAACGTCTATATAATGGCAGACAAGCAAAAAAACGGCATCAAGGTGAA
TTTTAAGATACGACATAATATCGAGGATGGGAGCGTCCAGTTGGCCGATCACTACCAACAGAATACTC
CCATTGGGGACGGGCCCGTGCTACTTCCAGATAATCACTACCTCTCATACCAGAGCGCATTATTCAAA
GACCCGAATGAAAAACGAGACCATATGGTTCTTTTGGAGTTTCTAACAGCCGCAGGGATCACCGAAGG
GATGAACGAGCTATATAAG

>roGFP_cds
ATGGTGAGCAAGGGCGAGGAGCTGTTCACCGGGGTGGTGCCCATCCTGGTCGAGCTGGACGGCGACG
TAAACGGCCACAAGTTCAGCGTGTCCGGCGAGGGCGAGGGCGATGCCACCTACGGCAAGCTGACCCT
GAAGTTCATCTCCACCACCGGCAAGCTGCCCGTGCCCTGGCCCACCCTCGTGACCACCCTGACCTACG
GCGTGCAGTGCTTCAGCCGCTACCCCGACCACATGAAGCAGCACGACTTCTTCAAGTCCGCCATGCCC
GAAGGCTACGTCCAGGAGCGCACCATCTTCTTCAAGGACGACGGCAACTACAAGACCCGCGCCGAGG
TGAAGTTCGAGGGCGACACCCTGGTGAACCGCATCGAGCTGAAGGGCATCGACTTCAAGGAGGACGG
CAACATCCTGGGGCACAAGCTGGAGTACAACTACAACTGCCACAACGTCTATATCATGGCCGACAAGC
AGAAGAACGGCATCAAGGTGAACTTCAAGATCCGCCACAACATCGAGGACGGCAGCGTGCAGCTCGC
CGACCACTACCAGCAGAACACCCCCATCGGCGACGGCCCCGTGCTGCTGCCCGACAACCACTACCTGA
GCACCTGCTCCGCCCTGAGCAAAGACCCCAACGAGAAGCGCGATCACATGGTCCTGCTGGAGTTCGTG
ACCGCCGCCGGGATCACTCTCGGCATGGACGAGCTGTACAAG

>BS1 sgRNA
GCAGCCCTCCAAAAAATGGCGTTTTAGAGCTAGAAATAGCAAGTTAAAATAAGGCTAGTCCGTTATCA
ACTTGAAAAAGTGGCACCGAGTCGGTGC

>BS1 Dark GFP cds
GCAGCCCTCCAAAAAATGGCCGGACACCACCACCACCACCACTGTCTAGAGGATCAATTCGATCCCCA
TGGTGAGCAAGGGCGAGGAGCTGTTCACCGGGGTGGTGCCCATCCTGGTCGAGCTGGACGGCGACGT
AAACGGCCACAAGTTCAGCGTGTCCGGCGAGGGCGAGGGCGATGCCACCTACGGCAAGCTGACCCTG
AAGTTCATCTGCACCACCGGCAAGCTGCCCGTGCCCTGGCCCACCCTCGTGACCACCTTCACCTACGGC
GTGCAGTGCTTCAGCCGCTACCCCGACCACATGAAGCAGCACGACTTCTTCAAGTCCGCCATGCCCGA
AGGCTACGTCCAGGAGCGCACCATCTTCTTCAAGGACGACGGCAACTACAAGACCCGCGCCGAGGTG
AAGTTCGAGGGCGACACCCTGGTGAACCGCATCGAGCTGAAGGGCATCGACTTCAAGGAGGACGGCA
ACATCCTGGGGCACAAGCTGGAGTACAACTACAACAGCCACAACGTCTATATCATGGCCGACAAGCA
GAAGAACGGCATCAAGGTGAACTTCAAGATCCGCCACAACATCGAGGACGGCAGCGTGCAGCTCGCC
GACCACTACCAGCAGAACACCCCCATCGGCGACGGCCCCGTGCTGCTGCCCGACAACCACTACCTGAG

CACCCAGTCCGCCCTGAGCAAAGACCCCAACGAGAAGCGCGATCACATGGTCCTGCTGGAGTTCGTGA
CCGCCGCCGGGATCACTCTCGGCATGGACGAGCTGTACAAGTAA

>BS2 sgRNA
GCACTACACGCCGTAGGTGAGTTTTAGAGCTAGAAATAGCAAGTTAAAATAAGGCTAGTCCGTTATCA
ACTTGAAAAAGTGGCACCGAGTCGGTGC

>BS2 Dark GFP cds
TTACTTGTACAGCTCGTCCATGCCGTGAGTGATCCCGGCGGCGGTCACGAACTCCAGCAGGACCATGT
GATCGCGCTTCTCGTTGGGGTCTTTGCTCAGGGCGGACTGGGTGCTCAGGTAGTGGTTGTCGGGCAGC
AGCACGGGGCCGTCGCCGATGGGGGTGTTCTGCTGGTAGTGGTCGGCGAGCTGCACGCTGCCGTCCTC
GATGTTGTGGCGGATCTTGAAGTTCACCTTGATGCCGTTCTTCTGCTTGTCGGCCATGATATAGACGTT
GTGGCTGTTGTAGTTGTACTCCAGCTTGTGCCCCAGGATGTTGCCGTCCTCCTTGAAGTCGATGCCCTT
CAGCTCGATGCGGTTCACCAGGGTGTCGCCCTCGAACTTCACCTCGGCGCGGGTCTTGTAGTTGCCGTC
GTCCTTGAAGAAGATGGTGCGCTCCTGGACGTAGCCTTCGGGCATGGCGGACTTGAAGAAGTCGTGCT
GCTTCATGTGGTCGGGGTAGCGGCTGAAGCACTACACGCCGTAGGTGAAGGTGGTCACGAGGGTGGG
CCAGGGCACGGGCAGCTTGCCGGTGGTGCAGATGAACTTCAGGGTCAGCTTGCCGTAGGTGGCATCGC
CCTCGCCCTCGCCGGACACGCTGAACTTGTGGCCGTTTACGTCGCCGTCCAGCTCGACCAGGATGGGC
ACCACCCCGGTGAACAGCTCCTCGCCCTTGCTCACCAT
