# Supplementary material for: Biological and Molecular Components for Genetically Engineering Biosensors in Plants
Source: Biodes Res. 2022 Nov 9;2022:9863496. doi: 10.34133/2022/9863496 (PMC10521658; doi:10.34133/2022/9863496)
Supplement: Supplementary Materials — Coding sequences for listed biosensors are provided in supplemental data 1-supplemental data 5. [file 9863496.f1.zip › Supplemental data 2 Sequences for biosensors in Table 2.pdf]

>G-GECO cds (Note: The cds sequence is kindly provided by Dr. Kevin Richard Cope, the first of author of the paper entitled “The ectomycorrhizal fungus *Laccaria bicolor* produces lipochitooligosaccharides and uses the common symbiosis pathway to colonize *Populus*”.)
ATGCCTAAGAAGAAGAGAAAGGTTGGAGGTATGGTCGACTCATCACGTCGTAAGTGGAATAAGACAG
GTCACGCAGTCAGAGCTATAGGTCGGCTGAGCTCACTCGAGAACGTCTATATCAAGGCCGACGAGCAG
AAGAACGGCATCAAGGCGTACTTCAAGATCCGCCACAACATCGAGGGCGGCGGCGTGCAGCTCGCCT
ACCACTACCAGCAGAACACCCCCATCGGCGACGGCCCCGTGCTGCTGCCCGACAACCACTACCTGAGC
GTGCAGTCCATGCTTTCGAAAGACCCCAACGAGAAGCGCGATCACATGGTCCTGCTGGAGTTCGTGAC
CGCCGCCGGGATCACTCTCGGCATGGACGAGCTGTACAAGGGCGGTACCGGAGGGAGCGAATCCATG
GTGAGCAAGGGCGAGGAGCTGTTCACCGGGGTGGTGCCCATCCAGGTCGAGCTGGACGGCGACGTAA
ACGGCCACAAGTTCAGCGTGTCCGGCGAGGGTGAGGGCGATGCCACCTACGGCAAGCTGACCCTGAA
GTTCATCTGCACCACCGGCAAGCTGCCCGTGCCCTGGCCCACCCTCGTGACCACCCTGACCTACGGCGT
GCAGTGCTTCAGCCGCTACCCCGACCACATGAAGCAGCACGACTTCTTCAAGTCCGCCATGCCCGAAG
GCTACATCCAGGAGCGCACCATCTTCTTCAAGGGCGACGGCAACTACAAGACCCGCGCCGAGGTGAA
GTTCGAGGGCGACACCCTGGTGAACCGCATCGAGCTGAAGGGCATCGACTTCAAGGAGGACGGCAAC
ATCCTGGGGCACAAGCTGGAGTACAACACGCGTGACCAACTGACTGAAGAGCAGATCGCAGAATTTA
AAGAGGCTTTCTCCCTATTTGACAAGGACGGGGATGGGACGATAACAACCAAGGAGCTGGGGACGGT
GATGCGGTCACTGGGGCAGAACCCCACAGAAGCAGAGCTGCAGGACATGATCAATGAAGTAGATGCC
GACGGTGACGGCACAATCGACTTCCCTGAGTTCCTGACAATGATGGCAAGAAAAATGAAAGACACAG
ACAGTGAAGAAGAAATTAGAGAAGCGTTCCGTGTGTTTGATAAGGACGGCAATGGCTACATCGGCGC
AGCAGAGCTTCGCCACGTGATGACAAACCTTGGAGAGAAGTTAACAGATGAAGAGGTTGATGAAATG
ATCAGGGTAGCAGACATCGATGGGGATGGTCAGGTAAACTACGAAGAGTTTGTACAAATGATGACAG
CGAAGTAG

> CALWY_NB Cerulean cds in green, Citrine cds in purple, Atox1-WD4 cds in grey.
ATGGTGAGCAAGGGCGAGGAGCTGTTCACCGGGGTGGTGCCCATCCTGGTCGAGCTGGACGGCGACG
TAAACGGCCACAAGTTCAGCGTGTCCGGCGAGGGCGAGGGCGATGCCACCTACGGCAAGCTGACCCT
GAAGTTCATCTGCACCACCGGTAAGCTGCCCGTGCCCTGGCCCACCCTCGTGACCACCCTGACCTGGG
GCGTGCAGTGCTTCGCCCGCTACCCCGACCACATGAAGCAGCACGACTTCTTCAAGTCCGCCATGCCC
GAAGGCTACGTCCAGGAGCGCACCATCTTCTTCAAGGACGACGGCAACTACAAGACCCGCGCCGAGG
TGAAGTTCGAGGGCGACACCCTGGTGAACCGCATCGAGCTGAAGGGCATCGACTTCAAGGAGGACGG
CAACATCCTGGGGCACAAGCTGGAGTACAACGCCATCAGCGACAACGTCTATATCACCGCCGACAAG
CAGAAGAACGGCATCAAGGCCAACTTCAAGATCCGCCACAACATCGAGGACGGCAGCGTGCAGCTCG
CCGACCACTACCAGCAGAACACCCCCATCGGCGACGGCCCCGTGCTGCTGCCCGACAACCACTACCTG
AGCACCCAGTCCGCCCTGTTCAAAGACCCCAACGAGAAGCGCGATCACATGGTCCTGCTGGAGTTCCT
GACCGCCGCCGGGATCACTCTCGGCATGGACGAGCTGTACAAGTCCGGAATGCCGAAGCACGAGTTCT
CTGTGGACATGACCAGTGGAGGCAGTGCTGAAGCTGTCTCTCGGGTCCTCAATAAGCTTGGAGGAGTT
AAGTATGACATTGACCTGCCCAACAAGAAGGTCTGCATTGAATCTGAGCACAGCATGGACACTCTGCT
TGCAACCCTGAAGAAAACAGGAAAGACTGTTTCCTACCTTGGCCTTGAGCTCATTCGTGGCGGATCCG
GCGGAAGCGGCGGATCCGGCGGTAGCGGCGGATCCGGCGGCTCCGGCGGATCCGGCGGCAGCGGCGG
ATCCGGTGGAAGCGGTGGATCCGGTGGTAGCGGTGGATCCGGTGGAAGCGGTGGATCCGGTGGTAGC
GGTGGATCCGGGGGTCCGCGGATGCAGGGCACATGCAGTACCACTCTGATTGCCATTGCCGGCATGAC
CAGTGCATCCAGTGTCCATTCCATTGAAGGCATGATCTCCCAACTGGAAGGGGTGCAGCAAATATCGG
TGTCTTTGGCCGAAGGGACTGCAACAGTTCTTTATAATCCCGCTGTAATTAGCCCAGAAGAACTCAGA
GCTGCTATAGAAGACATGGGATTTGAGGCTTCAGTCTCGGTACCCATGGTGAGCAAGGGCGAGGAGCT
GTTCACCGGGGTGGTGCCCATCCTGGTCGAGCTGGACGGCGACGTAAACGGCCACAAGTTCAGCGTGT
CCGGCGAGGGCGAGGGCGATGCCACCTACGGCAAGCTGACCCTGAAGTTCATCTGCACCACCGGCAA
GCTGCCCGTGCCCTGGCCCACCCTCGTGACCACCTTCGGCTACGGCCTGATGTGCTTCGCCCGCTACCC
CGACCACATGAAGCAGCACGACTTCTTCAAGTCCGCCATGCCCGAAGGCTACGTCCAGGAGCGCACCA
TCTTCTTCAAGGACGACGGCAACTACAAGACCCGCGCCGAGGTGAAGTTCGAGGGCGACACCCTGGTG
AACCGCATCGAGCTGAAGGGCATCGACTTCAAGGAGGACGGCAACATCCTGGGGCACAAGCTTGAGT
ACAACTACAACAGCCACAACGTCTATATCATGGCCGACAAGCAGAAGAACGGCATCAAGGTGAACTT
CAAGATCCGCCACAACATCGAGGACGGCAGCGTGCAGCTCGCCGACCACTACCAGCAGAACACCCCC
ATCGGCGACGGCCCCGTGCTGCTGCCCGACAACCACTACCTGAGCTACCAGTCCGCCCTGTTCAAAGA
CCCCAACGAGAAGCGCGATCACATGGTCCTGCTGGAGTTCCTGACCGCCGCCGGGATCACTCTCGGCA
TGGACGAGCTGTACAAGTAA

> FLIPPi-200u cds
CATCATCATCATCATCATGGTATGGCTAGCATGACTGGTGGACAGCAAATGGGTCGGGATCTGTACGA
CGATGACGATAAGGATCCGGGCCGCATGGTGAGCAAGGGCGAGGAGCTGTTCACCGGGGTGGTGCCC
ATCCTGGTCGAGCTGGACGGCGACGTAAACGGCCACAAGTTCAGCGTGTCCGGCGAGGGCGAGGGCG
ATGCCACCTACGGCAAGCTGACCCTGAAGTTCATCTGCACCACCGGCAAGCTGCCCGTGCCCTGGCCC
ACCCTCGTGACCACCCTGACCTGGGGCGTGCAGTGCTTCAGCCGCTACCCCGACCACATGAAGCAGCA
CGACTTCTTCAAGTCCGCCATGCCCGAAGGCTACGTCCAGGAGCGCACCATCTTCTTCAAGGACGACG
GCAACTACAAGACCCGCGCCGAGGTGAAGTTCGAGGGCGACACCCTGGTGAACCGCATCGAGCTGAA
GGGCATCGACTTCAAGGAGGACGGCAACATCCTGGGGCACAAGCTGGAGTACAACTACATCAGCCAC
AACGTCTATATCACCGCCGACAAGCAGAAGAACGGCATCAAGGCCAACTTCAAGATCCGCCACAACA
TCGAGGACGGCAGCGTGCAGCTCGCCGACCACTACCAGCAGAACACCCCCATCGGCGACGGCCCCGT
GCTGCTGCCCGACAACCACTACCTGAGCACCCAGTCCGCCCTGAGCAAAGACCCCAACGAGAAGCGC
GATCACATGGTCCTGCTGGAGTTCGTGACCGCCGCCGGGATCGGTACCGTAGGATTTCTAACAGCGAC
CTCGGCTCAAGCCCAAACCGTGCAAATCTCCGGGGCGGGCGCGACCTTTGCGGCTCCTTTGCTGCAAC
GTTGGTTTGACGCCTACAACCGCACCGTAGACCCCACTGTGCAAGTCAGCTATCAGTCTGTCGGTAGT
GGTGCTGGCCTAGAGCAGGTGATCAATGGCACTGTGGACTTCGGCGCTTCCGAGGCGCCTTTCTCCGG
TGCTCGCCTGGAGAGCTTCCGAGCTAAATACGGCTATGATCCCCTACAGTTGCCTCTGGCGGGAGGGG
CCATCGAGTTTGCCTATAACCTGCCCGGCATTGAAGACGGAGAGCTCATCCTGAAGCGGAAAACCTAC
TGCGGCATCGTGACCGGCGAGATCACTCGCTGGGACGACATTCGCATCAAGGCCGAGAACCCAGGTAT
AGCAAACAAGCTGCCACCCCTGGACATCACCTGGGTACACCGCTCTGATGGTTCTGCGACTACCTTTGT
GTTCACCAACCACATCAGAACTGTCTGCCCTAATTGGACAGCCGGTGCTGGTACTTCTGTCGAGTGGCC
TGTTGGTATTGGAGCCCAAGGGAATGAGGGCGTAGCCGCCACCATCAAGCAGGAGCCAGGGGCGATT
GGCTACGTGAACCAGTCCTATGCCAAGCTGGAAAAGATGGCCACTGCTCGCTTGGAAAACAAAGCGG
GCAACATTGTTGAGTTCTCGACTGAGGCAGCTACCTCGGCGCTGGATGCTCCCATTCCTGATGACTTTG
CGCTGTTGGTGCCCGACCCTGAAGGGCCAAATGACTACCCAATCGTGGGCTTGTTCTGGGTGATGCTG
TACCGCGAGTATCCCGATCAGCAGAAGCTGACCAAGCTGGTGGAGGCTCTGAAGTGGACCCAGGGGC
CAGAGGGTCAAGCCATCACCAAGGAGCTGGACTACATCCCTATGCCTGAGGCGGTTATCCAGCGGATC
TTTGCAGAGCTGGATTCCATCACCGTTAACGGTACCGGTGGAATGGTGAGCAAGGGCGAGGAGCTGTT
CACCGGGGTGGTGCCCATCCTGGTCGAGCTGGACGGCGACGTAAACGGCCACAAGTTCAGCGTGTCCG
GCGAGGGCGAGGGCGATGCCACCTACGGCAAGCTGACCCTGAAGTTCATCTGCACCACCGGCAAGCT
GCCCGTGCCCTGGCCCACCCTCGTGACCACCTTCGGCTACGGCCTGCAGTGCTTCGCCCGCTACCCCGA
CCACATGAAGCAGCACGACTTCTTCAAGTCCGCCATGCCCGAAGGCTACGTCCAGGAGCGCACCATCT
TCTTCAAGGACGACGGCAACTACAAGACCCGCGCCGAGGTGAAGTTCGAGGGCGACACCCTGGTGAA
CCGCATCGAGCTGAAGGGCATCGACTTCAAGGAGGACGGCAACATCCTGGGGCACAAGCTGGAGTAC
AACTACAACAGCCACAACGTCTATATCATGGCCGACAAGCAGAAGAACGGCATCAAGGTGAACTTCA
AGATCCGCCACAACATCGAGGACGGCAGCGTGCAGCTCGCCGACCACTACCAGCAGAACACCCCCAT
CGGCGACGGCCCCGTGCTGCTGCCCGACAACCACTACCTGAGCTACCAGTCCGCCCTGAGCAAAGACC
CCAACGAGAAGCGCGATCACATGGTCCTGCTGGAGTTCGTGACCGCCGCCGGGATCACTCTCGGCATG
GACGAGCTGTACAAGTAA

>ABSCUS1 cds
CCGGGCTTGTCTCCCTTCCCTTCCCGGTATCGGTTCATGGATTCGGTTAGATGGGAAACCGCCATCAGT
ACCAGGTCGTAATCCCACACACTGGCCATGCCGGCCGGCCCTGCGGAAACCTCTACGTGCCCGTCTGG
AAGCTCGTAGCGGATCACCTCGCCAGCTCGTCGGTCACGCTTCGACAGACGGAAAACGGCCACGTCCA
TGATGCTGCGACTATCGCGGGTGCCCACGTCATAGAGCATCGGAACGAAAAAATCTGGTTGCTCGTCG
CCCTTGGGCGGCTTCCTAATCGACGGCGCACCGGCTGCCGGCGGTTGCCGGGATTCTTTGCGGATTCG
ATCAGCGGCCGCTTGCCACGATTCACCGGGGCGTGCTTCTGCCTCGATGCGTTGCCGCTGGGCGGCCT
GCGCGGCCTTCAACTTCTCCACCAGGTCATCACCCAGCGCCGCGCCGATTTGTACCGGGCCGGATGGT
TTGCGACCGTCACGCCGATTCCTCGGGCTTGGGGGTTCCAGTGCCATTGCAGGGCCGGCAGACAACCC
AGCCGCTTACGCCTGGCCAACCGCCCGTTCCTCCACACATGGGGCATTCCACGGCGTCGGTGCCTGGTT
GTTCTTGATTTTCCATGCCGCCTCCTTTAGCCGCTAAAATTCATCTACTCATTTATTCATTTGCTCATTTA
CTCTGGTAGCTGCGCGATGTATTCAGATAGCAGCTCGGTAATGGTCTTGCCTTGGCGTACCGCGTACAT
CTTCAGCTTGGTGTGATCCTCCGCCGGCAACTGAAAGTTGACCCGCTTCATGGCTGGCGTGTCTGCCAG
GCTGGCCAACGTTGCAGCCTTGCTGCTGCGTGCGCTCGGACGGCCGGCACTTAGCGTGTTTGTGCTTTT
GCTCATTTTCTCTTTACCTCATTAACTCAAATGAGTTTTGATTTAATTTCAGCGGCCAGCGCCTGGACCT

CGCGGGCAGCGTCGCCCTCGGGTTCTGATTCAAGAACGGTTGTGCCGGCGGCGGCAGTGCCTGGGTAG
CTCACGCGCTGCGTGATACGGGACTCAAGAATGGGCAGCTCGTACCCGGCCAGCGCCTCGGCAACCTC
ACCGCCGATGCGCGTGCCTTTGATCGCCCGCGACACGACAAAGGCCGCTTGTAGCCTTCCATCCGTGA
CCTCAATGCGCTGCTTAACCAGCTCCACCAGGTCGGCGGTGGCCCATATGTCGTAAGGGCTTGGCTGC
ACCGGAATCAGCACGAAGTCGGCTGCCTTGATCGCGGACACAGCCAAGTCCGCCGCCTGGGGCGCTCC
GTCGATCACTACGAAGTCGCGCCGGCCGATGGCCTTCACGTCGCGGTCAATCGTCGGGCGGTCGATGC
CGACAACGGTTAGCGGTTGATCTTCCCGCACGGCCGCCCAATCGCGGGCACTGCCCTGGGGATCGGAA
TCGACTAACAGAACATCGGCCCCGGCGAGTTGCAGGGCGCGGGCTAGATGGGTTGCGATGGTCGTCTT
GCCTGACCCGCCTTTCTGGTTAAGTACAGCGATAACCTTCATGCGTTCCCCTTGCGTATTTGTTTATTTA
CTCATCGCATCATATACGCAGCGACCGCATGACGCAAGCTGTTTTACTCAAATACACATCACCTTTTTA
GACGGCGGCGCTCGGTTTCTTCAGCGGCCAAGCTGGCCGGCCAGGCCGCCAGCTTGGCATCAGACAAA
CCGGCCAGGATTTCATGCAGCCGCACGGTTGAGACGTGCGCGGGCGGCTCGAACACGTACCCGGCCGC
GATCATCTCCGCCTCGATCTCTTCGGTAATGAAAAACGGTTCGTCCTGGCCGTCCTGGTGCGGTTTCAT
GCTTGTTCCTCTTGGCGTTCATTCTCGGCGGCCGCCAGGGCGTCGGCCTCGGTCAATGCGTCCTCACGG
AAGGCACCGCGCCGCCTGGCCTCGGTGGGCGTCACTTCCTCGCTGCGCTCAAGTGCGCGGTACAGGGT
CGAGCGATGCACGCCAAGCAGTGCAGCCGCCTCTTTCACGGTGCGGCCTTCCTGGTCGATCAGCTCGC
GGGCGTGCGCGATCTGTGCCGGGGTGAGGGTAGGGCGGGGGCCAAACTTCACGCCTCGGGCCTTGGC
GGCCTCGCGCCCGCTCCGGGTGCGGTCGATGATTAGGGAACGCTCGAACTCGGCAATGCCGGCGAACA
CGGTCAACACCATGCGGCCGGCCGGCGTGGTGGTGTCGGCCCACGGCTCTGCCAGGCTACGCAGGCCC
GCGCCGGCCTCCTGGATGCGCTCGGCAATGTCCAGTAGGTCGCGGGTGCTGCGGGCCAGGCGGTCTAG
CCTGGTCACTGTCACAACGTCGCCAGGGCGTAGGTGGTCAAGCATCCTGGCCAGCTCCGGGCGGTCGC
GCCTGGTGCCGGTGATCTTCTCGGAAAACAGCTTGGTGCAGCCGGCCGCGTGCAGTTCGGCCCGTTGG
TTGGTCAAGTCCTGGTCGTCGGTGCTGACGCGGGCATAGCCCAGCAGGCCAGCGGCGGCGCTCTTGTT
CATGGCGTAATGTCTCCGGTTCTAGTCGCAAGTATTCTACTTTATGCGACTAAAACACGCGACAAGAA
AACGCCAGGAAAAGGGCAGGGCGGCAGCCTGTCGCGTAACTTAGGACTTGTGCGACATGTCGTTTTCA
GAAGACGGCTGCACTGAACGTCAGAAGCCGACTGCACTATAGCAGCGGAGGGGTTGGATCAAAGTAC
TTTGATCCCGAGGGGAACCCTGTGGTTGGCATGCACATACAAATGGACGAACGGATAAACCTTTTCAC
GCCCTTTTAAATATCCGTTATTCTAATAAACGCTCTTTTCTCTTAGGTTTACCCGCCAATATATCCTGTC
AAACACTGATAGTTTAAACTGAAGGCGGGAAACGACAATCTGATCCAAGCTCAAGCTGCTCTAGCCAA
TACGCAAACCGCCTCTCCCCGCGCGTTGGCCGATTCATTAATGCAGCTGGCACGACAGGTTTCCCGACT
GGAAAGCGGGCAGTGAGCGCAACGCAATTAATGTGAGTTAGCTCACTCATTAGGCACCCCAGGCTTTA
CACTTTATGCTTCCGGCTCGTATGTTGTGTGGAATTGTGAGCGGATAACAATTTCACACAGGAAACAG
CTATGACCATGATTACG

>AuxSen Aquamarine cds in purple, mNeonGreen cds in green, TrpR cds in grey. (Note: CDS sequences are codon optimized for *Arabidopsis thaliana* from protein sequences.)

ATGGTCAGCAAAGGTGAGGAGTTATTTACGGGTGTTGTTCCGATCTTAGTCGAGTTGGACGGGGACGT
GAACGGGCACAAGTTCAGCGTTTCCGGAGAAGGGGAGGGAGATGCTACCTATGGAAGCTCACATTA
AAATTTATATGTACTACAGGCAAGTTGCCCGTTCCTTGGCCTACCTTAGTTACGACTTTGTCATGGGGG
GTACAATGTTTTTCTAGATATCCTGATCACATGAAACAGCATGATTTCTTCAAATCCGCGATGCCGGAG
GGATACGTTCAAGAGCGTACAATTTTTTTCAAGGACGATGGAAACTACAAGACTCGAGCCGAAGTCAA
GTTCGAAGGCGATACACTCGTAAACCGAATCGAACTTAAAGGAATAGACTTTAAGGAGGACGGAAAT
ATCCTGGGTCACAAGCTCGAGTACAACTACATCTCCGGAAATGTCTATATAACGGCAGACAAACAAAA
GAATGGTATAAAGGCCAATTTCAAAATAAGACACAACATTGAGGATGGTTCAGTTCAGCTAGCCGACC
ACTACCAGCAGAACACTCCCATTGGAGACGGACCCGTGCTCCTCCCTGATAATCATTATTTAAGTACTC
AATCCGCCTTATCCAAGGACCCGAACGAGAAGCGTGACCACATGGTCCTACTAGAGTTTGTGACCGCA
GCAGGCATAACACTGGGGATGGATGAATTGTACAAGGAGCCCAACAATCACCCTATTCAGCAGCGAT
GGCAGAACAGCGTCACCAGGAGTGGTTACGTTTTGTCGACCTGCTTAAGAATGCCTACCAAAACGATC
TCCATTTACCGTTGTTAAACCTGATGCTGACGCCAGATGAGCGCGAAGCGTTGGGGACTCGCGTGCGT
ATTGTCGAAGAGCTGTTGCGCGGCGAAATGAGCCAGCGTGAGTTAAAAAATGAACTCGGCGCAGGCA
TCGCGACGATTACGCGTGGATCTAACAGCCTGAAAGCCGCGCCCGTCGAGCTGCGCCAGTGGCTGGAA
GAGGTGTTGCTGAAAAGCGATAATGGTCTCTAAAGGGGAGGAGGATAATATGGCCAGTCTACCAGCA
ACGCACGAGCTGCACATCTTTGGTTCCATCAACGGGGTAGATTTCGATATGGTTGGTCAAGGTACAGG
TAACCCAAACGATGGATACGAGGAGCTCAACCTGAAGTCCACGAAGGGTGACCTACAATTTTCACCCT
GGATTCTTGTCCCGCACATCGGTTATGGATTCCATCAGTACCTTCCGTATCCCGATGGTATGTCCCCCTT
TCAGGCCGCAATGGTCGACGGATCTGGATACCAAGTGCACAGGACGATGCAGTTCGAGGACGGTGCA

AGCCTAACAGTTAATTACAGGTACACCTACGAAGGCAGCCACATCAAAGGCGAGGCGCAAGTTAAAG
GGACCGGGTTCCCCGCTGACGGTCCTGTTATGACAAACTCCTTGACCGCTGCCGACTGGTGTAGGTCA
AAGAAAACTTATCCCAATGATAAAACTATTATATCTACCTTTAAGTGGTCTTATACTACCGGAAATGGT
AAGCGATATAGGAGTACAGCACGAACCACTTATACCTTTGCCAAACCTATGGCTGCTAACTATCTGAA
GAACCAACCGATGTACGTTTTTCGAAAGACTGAACTTAAACATTCCAAAACCGAGCTCAATTTTAAGG
AGTGGCAGAAAGCGTTCACAGATGTGATGGGAATGGACGAACTGTACAAA

>SED1 mCerulean3 cds in purple, Citrine cds in green, AtLEA4-5 cds in grey. (Note: The cds sequences of florescence proteins are codon optimized for *Arabidopsis thaliana* from protein sequences. The AtLEA4-5 cds is from phytozome (https://phytozome-next.jgi.doe.gov/).)
ATGGTATCTAAAGGCGAGGAATTGTTTACTGGTGTGGTACCAATCTTAGTAGAACTAGACGGGGATGT
AAATGGACATAAATTTAGTGTTTCTGGCGAGGGTGAAGGGGATGCTACTTATGGCAAATTGACACTCA
AGTTTATATGCACCACAGGGAAGTTACCAGTCCCCTGGCCCACCTTGGTTACGACATTGTCATGGGGG
GTTCAGTGTTTCGCTAGGTACCCCGATCACATGAAACAACACGATTTCTTCAAGAGTGCTATGCCCGA
GGGTTATGTCCAAGAGCGAACGATTTTTTTCAAGGACGACGGTAACTACAAGACGCGTGCTGAAGTGA
AATTTGAAGGGGACACACTGGTAAACCGAATTGAGCTAAAGGGGATTGATTTCAAGGAGGACGGTAA
CATACTCGGTCATAAACTCGAATATAACGCCATTCACGGTAACGTTTACATCACTGCTGACAAACAGA
AGAACGGGATTAAAGCCAACTTCGGGCTAAATTGTAACATTGAGGATGGTAGTGTGCAACTCGCAGAC
CACTATCAACAGAACACGCCAATTGGTGATGGACCAGTCTTATTACCAGACAACCACTACTTGTCTAC
GCAAAGCAAGTTGAGCAAAGACCCTAATGAGAAGAGAGACCATATGGTACTTTTGGAATTTGTTACAG
CGGCGGGGATAACGCTAGGCATGGATGAACTCTATAAGCTATGGCTATATCTATGGGACATGCTCGGT
GAGCAAAACAATAAACAAAAGTAATCAGAAGATATCTTTGTAACATCTTTGAATTTCGCTAAAGGAAA
AGAGAGAGATTTGGTAAAAATGCAGTCGATGAAAGAAACAGCTTCGAATATTGCAGCTTCTGCAAAA
TCTGGCATGGACAAAACCAAAGCTACCTTGGAGGAAAAGGCGGAGAAGATGAAGACACGAGACCCTG
TTCAGAAACAGATGGCTACACAGGTTAAAGAAGATAAGATCAATCAAGCTGAGATGCAGAAGAGAGA
AACGCGTCAGCACAACGCGGCCATGAAAGAAGCGGCTGGAGCCGGAACCGGTTTAGGTTTGGGGACG
GCCACTCACTCGACCACTGGACAAGTCGGACACGGCACTGGGACACATCAGATGTCGGCTCTGCCTGG
TCACGGAACGGGACAACTGACCGACCGCGTTGTGGAGGGCACGGCTGTGACCGACCCGATTGGAAGG
AACACTGGAACTGGTCGGACAACCGCTCATAACACTCACGTTGGTGGTGGTGGTGCCACCGGGTACGG
AACCGGCGGGGGATATACTGGATAAACGGTGTTGTTCACTTGTTCTGTTATTTGCTCTGTTTTTGTTGA
ATCGAAATTTCGTGGTTTTCTGTCTTACTTTCGTGTTCTGTTTTTTTTTTTCTTTTTCTTGTGCTTCTGAA
TAATTGTAGGACTTCGATCTCATCTAGGTTTTTGTTGTTGGATGATAGTCTTACTTTATGAGTAGAATGT
TCATGCAATGTAACAGTAGTAAAATTATCCGTTAACAATATTGTGCCCATGCAACTTAACAATAACAA
TACATTGCTAACTTTTTGAACTTTGAAGAGAAACATTGCTAATGCTAAGTTTTTGATGGTCTCCAAAGG
AGAGGAGCTTTTCACCGGAGTGGTGCCCATTCTCGTAGAGCTAGACGGGGATGTAAATGGGCACAAGT
TTTCTGTATCTGGAGAAGGCGAAGGAGATGCTACTTATGGGAAATTAACCTTGAAGTTTATATGCACG
ACTGGAAAGCTTCCTGTTCCCTGGCCTACTTTAGTGACTACGTTCGGCTATGGTCTAATGTGTTTCGCC
CGTTATCCTGACCATATGAAGCAGCATGACTTTTTCAAGAGCGCAATGCCTGAAGGTTATGTTCAGGA
ACGTACCATTTTTTTTAAAGATGACGGGAATTATAAAACTAGAGCAGAGGTCAAATTTGAAGGCGACA
CGTTAGTAAACCGAATCGAGCTGAAAGGGATAGACTTCAAAGAGGACGGAAATATTTTAGGGCACAA
ACTTGAATACAACTATAACTCACACAATGTGTACATAATGGCAGACAAACAGAAGAACGGGATAAAA
GTTAATTTCAAGATTCGACACAATATTGAAGATGGCTCCGTGCAACTGGCAGATCATTACCAGCAAAA
CACTCCAATTGGTGACGGTCCTGTACTACTACCAGATAATCATTATCTCAGTTATCAGTCAGCACTCAG
TAAAGATCCGAACGAGAAGAGGGATCATATGGTCTTATTAGAATTTGTAACGGCTGCAGGGATTACAT
TGGGCATGGACGAGCTTTATAAA
