# Supplementary material for: Biological and Molecular Components for Genetically Engineering Biosensors in Plants
Source: Biodes Res. 2022 Nov 9;2022:9863496. doi: 10.34133/2022/9863496 (PMC10521658; doi:10.34133/2022/9863496)
Supplement: Supplementary Materials — Coding sequences for listed biosensors are provided in supplemental data 1-supplemental data 5. [file 9863496.f1.zip › Supplemental data 3 Sequences for biosensors in Table 3.pdf]

>RD29A promoter
TAGGTAGAACTTATATACATTATATTGTAATTTTTTGTAACAAAATGTTTTTATTATTATTATAGAATTT
TACTGGTTAAATTAAAAATGAATAGAAAAGGTGAATTAAGAGGAGAGAGGAGGTAAACATTTTCTTCT
ATTTTTTCATATTTTCAGGATAAATTATTGTAAAAGTTTACAAGATTTCCATTTGACTAGTGTAAATGA
GGAATATTCTCTAGTAAGATCATTATTTCATCTACTTCTTTTATCTTCTACCAGTAGAGGAATAAACAA
TATTTAGCTCCTTTGTAAATACAAATTAATTTTCGTTCTTGACATCATTCAATTTTAATTTTACGTATAA
AATAAAAGATCATACCTATTAGAACGATTAAGGAGAAATACAATTCGAATGAGAAGGATGTGCCGTTT
GTTATAATAAACAGCCACACGACGTAAACGTAAAATGACCACATGATGGGCCAATAGACATGGACCG
ACTACTAATAATAGTAAGTTACATTTTAGGATGGAATAAATATCATACCGACATCAGTTTGAAAGAAA
AGGGAAAAAAAGAAAAAATAAATAAAAGATATACTACCGACATGAGTTCCAAAAAGCAAAAAAAAA
GATCAAGCCGACACAGACACGCGTAGAGAGCAAAATGACTTTGACGTCACACCACGAAAACAGACGC
TTCATACGTGTCCCTTTATCTCTCTCAGTCTCTCTATAAACTTAGTGAGACCCTCCTCTGTTTTACTC

>DR5_V2 promoter
CCGACAAAAGGCCGACAAAAGGCCGACAAAAGGCCGACAAAAGGCCGACAAAAGGCCGACAAAAGG
CCGACAAAAGGCCGACAAAAGGCCGACAAAAGGCCGACAAAAGGGGGCAGGCCTCGATAAGCTTGA
TATCGAATTAATTCCTGCAGCCCCGCAAGACCCTTCCTCTATATAAGGAAGTTCATTTCATTTGGAGAG
G

>TCS promoter
AAAATCTACAAAATCTTTTTGGATTTTGTGGATTTTCTAGCGCAAGACCCTTCCTCTATATAAGGAAGT
TCATTTCATTTGGAGAGGTATTTTTACAACAATTACCAACAACAACAAACAACAAACAACATTACAAT
TACTATTTACAATTAC

>FLS2 promoter
GCTTTGAAGACGTGTTAATGAATTAAACTGATTAGACCTAAAACCAAATATTTAGAAAGAAAAAAATA
TGATAATTGTTATGTTATTTAATCAATAGTCAAATACTATGTTGAGTACCAATCATTTCAGTTTCAACG
ATTGCTACTCTTTTGACCAATAACAAAAAGGTTTTTATTTTTCATTTCTCAAACTCCATTTTTGTTTGTTT
TTAGCATAAAAACAGCTAGAATGTGAGTTTCATATGTATTATATACTATAAGAAATCATATAAATTAA
CGACTAGAACATATTGTTTTCATTCTAGTGGTATATCTGAGTTTGACTAGAACGTTTGGAGGCAGATTA
AAGTGATTTCGCCTTGTCAAAACTACTTTGTATATGTTATCAATCAATCGCTCAAAACTAAATCGGAAA
AAAACTACATAAATAAGATATGGAAAATGATACTAACACAAACTAGTAAGTAAGTAGTCACTACATA
TTCTGGTCCCATGAATATGGCTTACATAGTACATACATTGTGAGATTAAATGAATATGATATATAATAC
AAGCATATAAGCATGTGCATGACTATGAAGTGGGAATACATTTAGTGCCTTTATATGCAACTTGACAG
AATTACCAATTATGTGAATAGTGAAACTTATATTGCGTCTTGGCCTTTTCACATCCGTGACATGATCCC
TCAAATCTATATCTTCTTGCGGCATATGCTTATATATAAACATATGACTTCGTCTATAGATTT

>SD18-1 promoter
GCTCATATGCTCATATGCTCATATGCTCATATGCTCATATGCTCATATGCAAGACCCTTCCTCTATATA
AGGAAGTTCATTTCATTTGGAGAGGTATTTTTACAACAATTACCAACAACAACAAACAACAAACAACA
TTACAATTACTATTTACAATTAC

>SS16-1 promoter
CAGATCAGATCAGATCAGATCAGATCAGATCAGATCAGATGCAAGACCCTTCCTCTATATAAGGAAGT
TCATTTCATTTGGAGAGGTATTTTTACAACAATTACCAACAACAACAAACAACAAACAACATTACAAT
TACTATTTACAATTAC

>SCN-1 promoter
TAAAGTTAAAGTTAAAGTTAAAGTTAAAGTTAAAGTGCAAGACCCTTCCTCTATATAAGGAAGTTCATTTCATT
TGGAGAGGTATTTTTACAACAATTACCAACAACAACAAACAACAAACAACATTACAATTACTATTTAC
AATTAC

>ER promoter

CTCGACACGCTCGACACGCTCGACACGCTCGACACGCTCGACACGCTCGACACGGCAAGACCCTTCCT
CTATATAAGGAAGTTCATTTCATTTGGAGAGGTATTTTTACAACAATTACCAACAACAACAAACAACA
AACAACATTACAATTACTATTTACAATTAC
