# Supplementary material for: Biological and Molecular Components for Genetically Engineering Biosensors in Plants
Source: Biodes Res. 2022 Nov 9;2022:9863496. doi: 10.34133/2022/9863496 (PMC10521658; doi:10.34133/2022/9863496)
Supplement: Supplementary Materials — Coding sequences for listed biosensors are provided in supplemental data 1-supplemental data 5. [file 9863496.f1.zip › Supplemental data 4 Sequences for biosensors in Table 4.pdf]

>DELLA_GFP DELLA cds in purple, GFP cds in green
ATAACCTTCCTCTCTATTTTTACAATTTATTTTGTTATTAGAAGTGGTAGTGGAGTGAAAAACAAATC
CTAAGCAGTCCTAACCGATCCCCGAAGCTAAAGATTCTTCACCTTCCCAAATAAAGCAAAACCTAGAT
CCGACATTGAAGGAAAAACCTTTTAGATCCATCTCTGAAAAAAAACCAACCATGAAGAGAGATCATC
ATCATCATCATCATCAAGATAAGAAGACTATGATGATGAATGAAGAAGACGACGGTAACGGCATGGA
TGAGCTTCTAGCTGTTCTTGGTTACAAGGTTAGGTCATCCGAAATGGCTGATGTTGCTCAGAAACTCGA
GCAGCTTGAAGTTATGATGTCTAATGTTCAAGAAGACGATCTTTCTCAACTCGCTACTGAGACTGTTCA
CTATAATCCGGCGGAGCTTTACACGTGGCTTGATTCTATGCTCACCGACCTTAATCCTCCGTCGTCTAA
CGCCGAGTACGATCTTAAAGCTATTCCCGGTGACGCGATTCTCAATCAGTTCGCTATCGATTCGGCTTC
TTCGTCTAACCAAGGCGGCGGAGGAGATACGTATACTACAAACAAGCGGTTGAAATGCTCAAACGGC
GTCGTGGAAACCACTACAGCGACGGCTGAGTCAACTCGGCATGTTGTCCTGGTTGACTCGCAGGAGAA
CGGTGTGCGTCTCGTTCACGCGCTTTTGGCTTGCGCTGAAGCTGTTCAGAAAGAGAATCTGACTGTAGC
GGAAGCTCTGGTGAAGCAAATCGGATTCTTAGCCGTTTCTCAAATCGGAGCGATGAGAAAAGTCGCTA
CTTACTTCGCCGAAGCTCTCGCGCGGCGGATTTACCGTCTCTCTCCGTCGCAGAGTCCAATCGACCACT
CTCTCTCCGATACTCTTCAGATGCACTTCTACGAGACTTGTCCTTATCTCAAGTTCGCTCACTTCACGGC
GAATCAAGCGATTCTCGAAGCTTTTCAAGGGAAGAAAAGAGTTCATGTCATTGATTTCTCTATGAGTC
AAGGTCTTCAATGGCCGGCGCTTATGCAGGCTCTTGCGCTTCGACCTGGTGGTCCTCCTGTTTTCCGGT
TAACCGGAATTGGTCCACCGGCACCGGATAATTTCGATTATCTTCATGAAGTTGGGTGTAAGCTGGCTC
ATTTAGCTGAGGCGATTCACGTTGAGTTTGAGTACAGAGGATTTGTGGCTAACACTTTAGCTGATCTTG
ATGCTTCGATGCTTGAGCTTAGACCAAGTGAGATTGAATCTGTTGCGGTTAACTCTGTTTTCGAGCTTC
ACAAGCTCTTGGGACGACCTGGTGCGATCGATAAGGTTCTTGGTGTGGTGAATCAGATTAAACCGGAG
ATTTTCACTGTGGTTGAGCAGGAATCGAACCATAATAGTCCGATTTTCTTAGATCGGTTTACTGAGTCG
TTGCATTATTACTCGACGTTGTTTGACTCGTTGGAAGGTGTACCGAGTGGTCAAGACAAGGTCATGTCG
GAGGTTTACTTGGGTAAACAGATCTGCAACGTTGTGGATGGTGAGCAAGGGCGAGGAGCTGTTCACCG
GGGTGGTGCCCATCCTGGTCGAGCTGGACGGCGACGTAAACGGCCACAAGTTCAGCGTGTCTGGCGAG
GGCGAGGGCGATGCCACCTACGGCAAGCTGACCCTGAAGTTCATCTGCACCACCGGCAAGCTGCCCGT
GCCCTGGCCCACCCTCGTGACCACCTTCACCTACGGCGTGCAGTGCTTCAGCCGCTACCCCGACCACAT
GAAGCAGCACGACTTCTTCAAGTCCGCCATGCCCGAAGGCTACGTCCAGGAGCGCACCATCTTCTTCA
AGGACGACGGCAACTACAAGACCCGCGCCGAGGTGAAGTTCGAGGGCGACACCCTGGTGAACCGCAT
CGAGCTGAAGGGCATCGACTTCAAGGAGGACGGCAACATCCTGGGGCACAAGCTGGAGTACAACTAC
AACAGCCACAACGTCTATATCATGGCCGACAAGCAGAAGAACGGCATCAAGGCGAACTTCAAGATCC
GCCACAACATCGAGGACGGCAGCGTGCAGCTCGCCGACCACTACCAGCAGAACACCCCCATCGGCGA
CGGCCCCGTGCTGCTGCCCGACAACCACTACCTGAGCACCCAGTCCGCCCTGAGCAAAGACCCCAACG
AGAAGCGCGATCACATGGTCCTGCTGGAGTTCGTGACCGCCGCCGGGATCACTCTCGGCATGGACGAG
CTGTACAAGTAA

>DII_VENUS DII cds in purple, VENUS cds in yellow, SV40 NLS in blue.
AAACAAAAAAGCTCGACCAAAGAAACATCATTCCTTTCCAATAACAGGGTTGAGGTAGCTCCAGTGGT
GGGATGGCCGCCGGTGAGATCATCCCGGAGAAACCTAACCCCAAGAAGAAGAGAAAGGTAGATCCCC
GGGCTGCAGGAATTAATTCGATCATGGTGAGCAAGGGCGAGGAGCTGTTCACCGGGGTGGTGCCCATC
CTGGTCGAGCTGGACGGCGACGTAAACGGCCACAAGTTCAGCGTGTCCGGCGAGGGCGAGGGCGATG
CCACCTACGGCAAGCTGACCCTGAAGCTGATCTGCACCACCGGCAAGCTGCCCGTGCCCTGGCCCACC
CTCGTGACCACCCTGGGCTACGGCCTGCAGTGCTTCGCCCGCTACCCCGACCACATGAAGCAGCACGA
CTTCTTCAAGTCCGCCATGCCCGAAGGCTACGTCCAGGAGCGCACCATCTTCTTCAAGGACGACGGCA
ACTACAAGACCCGCGCCGAGGTGAAGTTCGAGGGCGACACCCTGGTGAACCGCATCGAGCTGAAGGG
CATCGACTTCAAGGAGGACGGCAACATCCTGGGGCACAAGCTGGAGTACAACTACAACAGCCACAAC
GTCTATATCACCGCCGACAAGCAGAAGAACGGCATCAAGGCCAACTTCAAGATCCGCCACAACATCG
AGGACGGCGGCGTGCAGCTCGCCGACCACTACCAGCAGAACACCCCCATCGGCGACGGCCCCGTGCT
GCTGCCCGACAACCACTACCTGAGCTACCAGTCCGCCCTGAGCAAAGACCCCAACGAGAAGCGCGAT
CACATGGTCCTGCTGGAGTTCGTGACCGCCGCCGGGATCACTCTCGGCATGGACGAGCTGTACAAG

>Jas9_VENUS Jas9 cds in purple, VENUS cds in yellow, SV40 NLS in blue.
CCGCAGGCCCGTAAAGCCAGTTTAGCTAGATTCCTAGAAAAGCGAAAAGAACGTTTGATGAGTGCGAT
GCCTTACCCCAAGAAGAAGAGAAAGGTAGATCCCCGGGCTGCAGGAATTAATTCGATCATGGTGAGC
AAGGGCGAGGAGCTGTTCACCGGGGTGGTGCCCATCCTGGTCGAGCTGGACGGCGACGTAAACGGCC
ACAAGTTCAGCGTGTCCGGCGAGGGCGAGGGCGATGCCACCTACGGCAAGCTGACCCTGAAGCTGAT

CTGCACCACCGGCAAGCTGCCCGTGCCCTGGCCCACCCTCGTGACCACCCTGGGCTACGGCCTGCAGT
GCTTCGCCCGCTACCCCGACCACATGAAGCAGCACGACTTCTTCAAGTCCGCCATGCCCGAAGGCTAC
GTCCAGGAGCGCACCATCTTCTTCAAGGACGACGGCAACTACAAGACCCGCGCCGAGGTGAAGTTCG
AGGGCGACACCCTGGTGAACCGCATCGAGCTGAAGGGCATCGACTTCAAGGAGGACGGCAACATCCT
GGGGCACAAGCTGGAGTACAACTACAACAGCCACAACGTCTATATCACCGCCGACAAGCAGAAGAAC
GGCATCAAGGCCAACTTCAAGATCCGCCACAACATCGAGGACGGCGGCGTGCAGCTCGCCGACCACT
ACCAGCAGAACACCCCCATCGGCGACGGCCCCGTGCTGCTGCCCGACAACCACTACCTGAGCTACCAG
TCCGCCCTGAGCAAAGACCCCAACGAGAAGCGCGATCACATGGTCCTGCTGGAGTTCGTGACCGCCGC
CGGGATCACTCTCGGCATGGACGAGCTGTACAAG

> AtSMXL6_ Luciferase AtSMXL6 cds in purple, Luciferase cds in yellow,
ATGCCGACGCCGGTGACTACGGCGAGAGAATGCTTGACGGAAGAAGCTGCTCGTGCCCTCGACGATG
CTGTTGTTGTAGCTCGTCGGAGAAGCCACGCGCAGACGACGTCTCTTCATGCAGTTTCTGCTCTTTTAG
CTATGCCGTCGTCGATTCTCCGTGAAGTTTGCGTCTCACGCGCCGCTAGGAGTGTTCCTTACTCGTCGC
GACTTCAATTCCGAGCTCTTGAGCTCTGCGTCGGTGTATCTCTCGACAGGCTTCCGTCGTCGAAGTCTC
CGGCGACGGAAGAAGATCCACCGGTTTCGAATTCGCTCATGGCGGCGATCAAACGGTCTCAGGCGAA
CCAGAGACGGCATCCGGAGTCGTATCATCTTCAGCAGATCCACGCTAGTAACAACGGCGGGGGAGGA
TGCCAGACGACGGTTTTGAAAGTCGAATTGAAGTATTTCATACTCTCGATCCTTGACGATCCGATTGTG
AATCGGGTATTCGGAGAAGCTGGGTTTCGGAGCTCCGAAATTAAGCTCGATGTGCTTCACCCTCCGGT
AACACAACTTTCTTCCCGTTTCTCTAGAGGTCGTTGTCCGCCTCTCTTCCTCTGTAATCTTCCCAACTCA
GATCCGAATCGTGAGTTCCCGTTTAGTGGGAGCAGTGGTTTCGATGAAAATTCCCGGAGGATTGGAGA
AGTATTAGGCAGGAAAGATAAGAAGAACCCTCTGCTTATTGGTAACTGTGCTAATGAAGCTCTTAAAA
CGTTCACGGATTCGATCAACAGTGGGAAGTTAGGGTTTCTTCAGATGGATATTAGCGGATTAAGCTTG
ATTAGTATCGAGAAGGAGATTAGTGAGATTTTAGCCGATGGATCGAAAAACGAAGAAGAGATTCGAA
TGAAAGTGGATGACCTAGGAAGAACTGTAGAGCAAAGTGGCTCGAAATCGGGGATAGTGCTTAATCT
GGGAGAGCTCAAGGTTTTGACCAGTGAAGCCAATGCGGCTCTTGAGATTTTGGTGTCGAAGCTTTCGG
ATTTGCTGAAACATGAAAGTAAACAACTTTCGTTCATCGGATGTGTATCGAGTAATGAGACTTACACG
AAGCTTATTGATCGGTTTCCTACTATAGAGAAGGATTGGGACCTTCATGTTCTTCCAATCACAGCCTCT
ACTAAACCTTCGACTCAAGGGGTTTATCCCAAATCGAGCTTGATGGGATCCTTTGTTCCCTTTGGAGGG
TTCTTCTCATCAACATCAAATTTCAGAGTTCCATTGAGTAGCACAGTGAATCAGACGCTCTCTAGATGC
CACCTCTGCAACGAGAAGTATTTGCAAGAAGTAGCCGCTGTTCTCAAGGCCGGTTCGAGTCTTTCTCTG
GCTGACAAATGTTCCGAGAAGTTAGCCCCATGGCTACGGGCTATTGAGACCAAAGAAGACAAGGGAA
TAACAGGCAGCAGTAAGGCTTTAGATGACGCTAATACATCAGCCTCGCAAACCGCTGCTCTACAGAAG
AAATGGGACAACATATGCCAAAGTATCCATCACACTCCGGCGTTTCCTAAACTTGGTTTTCAGTCGGTG
AGTCCGCAGTTCCCAGTTCAGACTGAGAAGAGTGTGAGAACTCCTACAAGCTATTTGGAGACGCCTAA
ACTGCTGAATCCGCCAATCTCAAAGCCAAAACCTATGGAGGATCTTACGGCATCGGTGACTAACCGCA
CAGTGAGTTTGCCTTTGAGCTGTGTTACTACAGATTTTGGGTTGGGAGTAATCTATGCATCCAAAAACC
AGGAATCAAAAACAACGAGGGAGAAACCGATGCTGGTGACTCTAAACTCTTCTTTAGAACATACATAT
CAGAAAGATTTCAAGTCTCTCAGAGAAATACTCTCTCGTAAAGTTGCCTGGCAGACCGAAGCTGTAAA
TGCCATAAGCCAAATTATCTGCGGATGCAAAACCGACTCCACGCGAAGAAACCAAGCAAGCGGAATT
TGGCTGGCTCTTCTTGGACCCGATAAAGTGGGGAAGAAGAAAGTGGCGATGACTCTTTCTGAAGTCTT
CTTTGGTGGTAAAGTCAATTACATATGTGTAGATTTTGGGGCAGAGCATTGTTCCCTTGATGACAAATT
CAGAGGCAAAACAGTGGTGGATTACGTAACCGGTGAGTTATCTAGGAAACCACACTCTGTTGTTTTAC
TCGAAAACGTGGAAAAAGCTGAGTTCCCGGATCAGATGAGATTGTCTGAAGCTGTGAGTACGGGGAA
AATCCGTGATTTGCATGGAAGAGTGATTAGTATGAAAAATGTGATTGTTGTTGTGACGTCTGGGATTG
CCAAGGATAATGCCACTGACCATGTTATTAAACCTGTGAAGTTTCCTGAGGAGCAAGTTCTCAGCGCG
AGAAGCTGGAAACTGCAGATAAAGCTAGGAGATGCTACTAAATTTGGGGTAAATAAGAGAAAATATG
AGCTAGAAACAGCGCAACGTGCAGTGAAGGTGCAACGTTCATATCTGGATCTGAATCTTCCAGTGAAT
GAAACAGAATTTAGCCCTGATCATGAGGCAGAGGACAGGGACGCTTGGTTCGATGAATTCATTGAAA
AAGTAGATGGAAAAGTGACGTTCAAACCGGTTGATTTCGATGAGTTAGCCAAGAACATTCAAGAGAA
GATTGGTTCACATTTTGAGCGGTGCTTTGGATCCGAAACACATCTAGAACTTGATAAAGAAGTGATCC
TTCAGATTCTGGCGGCTTCATGGTCATCATTATCATCGGGCGAAGAAGAAGGGAGAACAATAGTTGAT
CAGTGGATGCAAACAGTTCTTGCTCGAAGCTTTGCTGAAGCAAAACAGAAGTACGGTTCGAATCCCAT
GTTGGGCGTGAAGCTGGTTGCTTCTTCTAGCGGCTTAGCTTCCGGAGTAGAATTGCCGGCGAAGGTGG
ATGTGATATGGTGAATGGAAGACGCCAAAAACATAAAGAAAGGCCCGGCGCCATTCTATCCGCTGGA
AGATGGAACCGCTGGAGAGCAACTGCATAAGGCTATGAAGAGATACGCCCTGGTTCCTGGAACAATT

GCTTTTACAGATGCACATATCGAGGTGGACATCACTTACGCTGAGTACTTCGAAATGTCCGTTCGGTTG
GCAGAAGCTATGAAACGATATGGGCTGAATACAAATCACAGAATCGTCGTATGCAGTGAAAACTCTCT
TCAATTCTTTATGCCGGTGTTGGGCGCGTTATTTATCGGAGTTGCAGTTGCGCCCGCGAACGACATTTA
TAATGAACGTGAATTGCTCAACAGTATGGGCATTTCGCAGCCTACCGTGGTGTTCGTTTCCAAAAAGG
GGTTGCAAAAAATTTTGAACGTGCAAAAAAAGCTCCCAATCATCCAAAAAATTATTATCATGGATTCT
AAAACGGATTACCAGGGATTTCAGTCGATGTACACGTTCGTCACATCTCATCTACCTCCCGGTTTTAAT
GAATACGATTTTGTGCCAGAGTCCTTCGATAGGGACAAGACAATTGCACTGATCATGAACTCCTCTGG
ATCTACTGGTCTGCCTAAAGGTGTCGCTCTGCCTCATAGAACTGCCTGCGTGAGATTCTCGCATGCCAG
AGATCCTATTTTTGGCAATCAAATCATTCCGGATACTGCGATTTTAAGTGTTGTTCCATTCCATCACGG
TTTTGGAATGTTTACTACACTCGGATATTTGATATGTGGATTTCGAGTCGTCTTAATGTATAGATTTGA
AGAAGAGCTGTTTCTGAGGAGCCTTCAGGATTACAAGATTCAAAGTGCGCTGCTGGTGCCAACCCTAT
TCTCCTTCTTCGCCAAAAGCACTCTGATTGACAAATACGATTTATCTAATTTACACGAAATTGCTTCTG
GTGGCGCTCCCCTCTCTAAGGAAGTCGGGGAAGCGGTTGCCAAGAGGTTCCATCTGCCAGGTATCAGG
CAAGGATATGGGCTCACTGAGACTACATCAGCTATTCTGATTACACCCGAGGGGGATGATAAACCGGG
CGCGGTCGGTAAAGTTGTTCCATTTTTTGAAGCGAAGGTTGTGGATCTGGATACCGGGAAAACGCTGG
GCGTTAATCAAAGAGGCGAACTGTGTGTGAGAGGTCCTATGATTATGTCCGGTTATGTAAACAATCCG
GAAGCGACCAACGCCTTGATTGACAAGGATGGATGGCTACATTCTGGAGACATAGCTTACTGGGACGA
AGACGAACACTTCTTCATCGTTGACCGCCTGAAGTCTCTGATTAAGTACAAAGGCTATCAGGTGGCTC
CCGCTGAATTGGAATCCATCTTGCTCCAACACCCCAACATCTTCGACGCTGGTGTCGCAGGTCTTCCCG
ACGATGACGCCGGTGAACTTCCCGCCGCCGTTGTTGTTTTGGAGCACGGAAAGACGATGACGGAAAAA
GAGATCGTGGATTACGTCGCCAGTCAAGTAACAACCGCGAAAAAGTTGCGCGGAGGAGTTGTGTTTGT
GGACGAAGTACCGAAAGGTCTTACCGGAAAACTCGACGCAAGAAAAATCAGAGAGATCCTCATAAAG
GCCAAGAAGGGCGGAAAGATCGCCGTGT
