# Supplementary material for: Biological and Molecular Components for Genetically Engineering Biosensors in Plants
Source: Biodes Res. 2022 Nov 9;2022:9863496. doi: 10.34133/2022/9863496 (PMC10521658; doi:10.34133/2022/9863496)
Supplement: Supplementary Materials — Coding sequences for listed biosensors are provided in supplemental data 1-supplemental data 5. [file 9863496.f1.zip › Supplemental data 5 Sequences for biosensors in Table 5.pdf]

>BZR1_VENUS BZR1 cds in purple, VENUS cds in yellow, SV40 NLS in blue.
ATGACTTCGGATGGAGCTACGTCGACATCAGCAGCTGCAGCTGCGGCGGCGGCAGCAGCGGCGAGGA
GGAAGCCGTCGTGGAGAGAAAGGGAGAATAATCGGAGGAGAGAAAGACGGAGAAGAGCTGTAGCTG
CGAAGATATACACTGGGCTTAGAGCTCAAGGTGATTATAATTTGCCTAAACATTGTGATAATAATGAA
GTCCTTAAAGCTCTTTGTGTTGAAGCTGGTTGGGTTGTTGAAGAAGATGGTACTACTTATCGCAAGGGA
TGCAAGCCTTTACCTGGTGAGATAGCTGGGACTTCATCTCGAGTAACTCCATATTCATCACAGAACCA
GAGCCCTCTTTCATCAGCCTTTCAAAGTCCCATCCCATCTTACCAAGTTAGCCCGTCTTCTTCATCATTC
CCGAGTCCTTCTCGCGGTGAACCAAATAACAACATGTCCTCTACATTCTTCCCTTTCCTCAGAAATGGT
GGCATTCCTTCTTCTCTTCCTTCCCTCAGAATCTCAAACAGTTGTCCAGTTACCCCACCGGTCTCATCGC
CGACTTCTAAGAACCCGAAACCGTTGCCTAACTGGGAATCTATCGCTAAGCAATCCATGGCCATTGCT
AAACAATCAATGGCGTCTTTTAATTATCCTTTCTATGCGGTTTCTGCACCTGCTAGTCCGACACATCGC
CACCAGTTTCATACCCCGGCTACTATACCTGAATGTGATGAGTCTGACTCTTCCACTGTTGATTCTGGT
CATTGGATAAGCTTTCAGAAGTTTGCACAACAACAGCCATTCTCTGCCTCTATGGTGCCAACCTCTCCT
ACCTTCAATCTTGTGAAACCTGCGCCTCAGCAGATGTCTCCAAATACTGCTGCCTTCCAAGAGATTGGT
CAAAGCTCTGAGTTTAAATTTGAGAATAGCCAAGTTAAACCCTGGGAAGGAGAGAGGATACATGATG
TGGGTATGGAGGATCTTGAGCTTACACTTGGAAATGGGAAGGCTCGTGGTTGACCCAAGAAGAAGAG
AAAGGTAGATCCCCGGGCTGCAGGAATTAATTCGATCATGGTGAGCAAGGGCGAGGAGCTGTTCACC
GGGGTGGTGCCCATCCTGGTCGAGCTGGACGGCGACGTAAACGGCCACAAGTTCAGCGTGTCCGGCG
AGGGCGAGGGCGATGCCACCTACGGCAAGCTGACCCTGAAGCTGATCTGCACCACCGGCAAGCTGCC
CGTGCCCTGGCCCACCCTCGTGACCACCCTGGGCTACGGCCTGCAGTGCTTCGCCCGCTACCCCGACCA
CATGAAGCAGCACGACTTCTTCAAGTCCGCCATGCCCGAAGGCTACGTCCAGGAGCGCACCATCTTCT
TCAAGGACGACGGCAACTACAAGACCCGCGCCGAGGTGAAGTTCGAGGGCGACACCCTGGTGAACCG
CATCGAGCTGAAGGGCATCGACTTCAAGGAGGACGGCAACATCCTGGGGCACAAGCTGGAGTACAAC
TACAACAGCCACAACGTCTATATCACCGCCGACAAGCAGAAGAACGGCATCAAGGCCAACTTCAAGA
TCCGCCACAACATCGAGGACGGCGGCGTGCAGCTCGCCGACCACTACCAGCAGAACACCCCCATCGG
CGACGGCCCCGTGCTGCTGCCCGACAACCACTACCTGAGCTACCAGTCCGCCCTGAGCAAAGACCCCA
ACGAGAAGCGCGATCACATGGTCCTGCTGGAGTTCGTGACCGCCGCCGGGATCACTCTCGGCATGGAC
GAGCTGTACAAG

>NLP7_sfGFP NLP7 cds in purple, sfGFP cds in green
ATGTGCGAGCCCGATGATAATTCCGCTAGAAACGGCGTCACTACTCAACCTTCGAGGTCAAGGGAGCT
TCTAATGGATGTTGACGACTTAGATCTTGACGGTTCATGGCCACTAGATCAAATCCCTTACTTATCCTC
ATCGAATCGCATGATTTCTCCGATTTTTGTCTCCTCTTCCTCTGAGCAGCCTTGCTCGCCTCTCTGGGCT
TTCTCCGACGGTGGAGGAAATGGTTTTCACCACGCAACCTCCGGTGGCGATGATGAGAAGATCAGCTC
TGTCTCCGGTGTTCCTTCTTTCCGTCTCGCCGAGTATCCTCTCTTCCTCCCTTACTCTTCTCCATCAGCAG
CTGAGAACACAACAGAGAAGCATAACAGTTTCCAGTTTCCGTCTCCATTGATGAGCCTAGTCCCACCA
GAGAACACAGACAACTACTGTGTGATCAAAGAGAGGATGACTCAGGCGCTTCGATACTTCAAAGAAT
CAACCGAACAACACGTTTTGGCTCAGGTCTGGGCTCCTGTGAGAAAGAATGGTCGTGATTTGCTGACG
ACTTTGGGTCAACCTTTTGTTCTTAATCCTAATGGTAATGGGCTTAATCAATACAGGATGATCTCTCTC
ACATATATGTTTTCTGTGGATAGTGAAAGTGACGTAGAGCTCGGACTCCCGGGTCGAGTTTTCCGTCAG
AAATTGCCTGAATGGACTCCAAATGTTCAGTACTATTCCAGCAAAGAATTCTCGCGGCTTGATCACGC
CTTGCACTACAACGTGCGTGGTACACTGGCCTTGCCTGTCTTTAATCCCTCTGGTCAGTCCTGCATAGG
TGTTGTGGAACTTATAATGACCTCAGAGAAGATTCACTATGCACCCGAAGTGGACAAAGTTTGCAAAG
CCCTTGAGGCGGTAAATCTGAAAAGCTCGGAAATACTTGATCACCAAACAACACAGATATGCAATGA
GAGTCGCCAAAACGCGCTTGCTGAGATTCTCGAAGTGCTGACAGTTGTATGTGAGACCCATAACTTGC
CTCTCGCTCAGACTTGGGTTCCATGTCAGCATGGGAGCGTTCTTGCCAATGGTGGCGGTCTAAAGAAA
AACTGCACCAGCTTTGACGGTAGCTGCATGGGTCAAATCTGCATGTCTACAACCGACATGGCCTGCTA
TGTCGTGGATGCTCATGTCTGGGGCTTTAGAGATGCCTGTCTTGAACACCATCTCCAGAAAGGCCAGG
GAGTCGCTGGACGAGCTTTTCTCAATGGTGGCTCATGTTTCTGCAGAGACATCACCAAGTTCTGCAAA
ACGCAGTACCCACTAGTCCATTATGCGCTCATGTTCAAGTTGACCACTTGTTTTGCAATATCTCTCCAG
AGCTCTTACACGGGCGACGACAGTTACATTCTTGAATTTTTTCTTCCTTCGAGTATAACAGACGACCAA
GAGCAAGATTTGCTGTTGGGTTCTATTTTGGTGACAATGAAAGAACATTTTCAGAGTCTGAGGGTTGC
ATCTGGGGTTGACTTTGGTGAAGATGACGACAAATTGTCTTTCGAGATCATCCAAGCATTACCGGACA
AGAAGGTTCATTCAAAAATAGAATCCATTCGAGTTCCCTTTTCTGGTTTTAAGTCAAATGCAACAGAG
ACGATGTTGATTCCTCAGCCTGTGGTTCAGTCTTCTGATCCAGTAAATGAGAAAATCAACGTGGCCACT

GTTAACGGTGTGGTTAAGGAGAAGAAGAAAACAGAGAAAAAGCGTGGGAAGACTGAGAAAACAATC
AGTCTAGATGTACTTCAGCAGTATTTCACTGGAAGTCTCAAAGACGCTGCAAAGAGCCTAGGAGTTTG
CCCGACGACAATGAAGCGAATTTGCAGGCAACACGGAATCTCGCGGTGGCCATCGAGGAAGATCAAG
AAAGTGAATCGTTCAATCACAAAGCTGAAACGAGTCATCGAATCTGTTCAAGGTACTGATGGAGGCCT
CGACCTGACTTCCATGGCCGTTAGTTCCATCCCTTGGACACACGGTCAAACATCAGCACAGCCACTAA
ACTCACCCAATGGTTCCAAACCACCTGAGCTACCAAACACCAATAATTCACCTAACCATTGGTCAAGT
GATCACAGTCCGAACGAGCCAAATGGTTCGCCTGAGTTACCACCAAGCAATGGTCACAAGCGATCACG
AACGGTGGATGAGAGCGCTGGGACTCCAACCTCTCATGGCTCATGTGACGGTAACCAATTAGATGAAC
CGAAAGTCCCAAATCAAGATCCGCTCTTCACGGTTGGTGGATCACCCGGGCTCCTTTTTCCACCTTATT
CTAGAGATCATGATGTATCTGCAGCTTCCTTCGCAATGCCGAACAGGCTTCTTGGTTCTATAGACCATT
TCCGAGGAATGCTCATTGAAGACGCTGGAAGTTCAAAAGATCTGAGAAATCTCTGCCCCACTGCAGCA
TTTGACGATAAGTTTCAAGACACAAACTGGATGAACAATGATAATAATAGCAACAACAACTTATACGC
TCCCCCAAAGGAAGAGGCCATTGCAAATGTTGCATGCGAACCATCAGGCTCAGAAATGAGAACGGTA
ACAATCAAAGCAAGTTACAAAGACGACATAATACGGTTCAGAATATCCTCGGGTTCAGGTATAATGGA
ATTGAAGGATGAAGTGGCTAAGAGGCTGAAAGTTGATGCAGGAACGTTCGATATCAAGTATCTTGACG
ATGATAACGAATGGGTTTTAATAGCTTGTGATGCTGATCTTCAAGAATGTCTCGAGATCCCTAGATCCT
CCCGCACGAAAATCGTAAGGCTCTTAGTTCATGATGTAACGACAAATCTAGGGAGCTCCTGCGAGAGC
ACTGGAGAATTGTGAATGAGCAAAGGCGAAGAACTGTTCACGGGGGTTGTACCCATTCTTGTAGAACT
GGATGGAGATGTCAATGGTCACAAGTTCAGCGTGAGGGGAGAAGGAGAAGGCGATGCCACAAACGGT
AAATTGACCCTTAAGTTCATATGCACGACCGGTAAACTACCTGTACCCTGGCCGACGCTGGTAACTAC
ATTAACCTATGGGGTACAATGCTTTTCCCGATACCCGGACCATATGAAGCGACACGACTTTTTCAAGTC
TGCTATGCCTGAAGGCTATGTGCAGGAGAGGACGATTTCCTTTAAGGACGACGGAACCTACAAAACGC
GTGCGGAGGTGAAGTTCGAAGGAGATACCCTCGTGAACCGAATCGAACTAAAGGGTATAGATTTTAA
GGAGGACGGAAACATATTGGGCCATAAATTGGAATATAATTTTAACAGTCATAACGTGTATATTACCG
CAGATAAGCAAAAGAATGGTATTAAGGCCAACTTCAAGATAAGGCATAACGTAGAAGACGGCTCTGT
CCAGTTAGCTGATCACTACCAACAGAACACTCCTATTGGCGATGGTCCCGTGCTATTGCCTGACAACC
ATTATCTATCTACTCAATCCGTATTAAGCAAGGACCCCAACGAAAAGCGAGATCACATGGTACTACTA
GAGTTCGTTACTGCGGCTGGGATCACGCATGGAATGGATGAACTCTACAAAGCTGCGAACGATGAAA
ATTATGCCCTAGCGGCT

>AtPH_CITRINE AtPH1 cds in purple, CITRINE cds in green.
TTCTGGTCAAACCCTGAGCGTTCTGGTTGGCTCACAAAGCAAGGCGATTACATCAAAACCTGGCGTCG
TCGTTGGTTCGTTCTCAAACGAGGGAAGCTTCTCTGGTTCAAAGATCAAGCCGCTGCTGGAATTCGTGG
ATCTACGCCGCGTGGTGTGATCTCCGTTGGTGATTGTCTCACCGTGAAAGGAGCTGAGGATGTTGTGA
ATAAGCCTTTTGCTTTTGAGCTATCTAGTGGTAGCTATACCATGTTCTTCATTGCTGATAATGAGAAGG
AGAAAGAAGAGTGGATTAATTCGATTGGAAGATCGATTGTGCAAATGGTGAGCAAGGGCGAGGAGCT
GTTCACCGGGGTGGTGCCCATCCTGGTCGAGCTGGACGGCGACGTAAACGGCCACAAGTTCAGCGTGT
CCGGCGAGGGCGAGGGCGATGCCACCTACGGCAAGCTGACCCTGAAGTTCATCTGCACCACCGGCAA
GCTGCCCGTGCCCTGGCCCACCCTCGTGACCACCTTCGGCTACGGCCTGATGTGCTTCGCCCGCTACCC
CGACCACATGAAGCAGCACGACTTCTTCAAGTCCGCCATGCCCGAAGGCTACGTCCAGGAGCGCACCA
TCTTCTTCAAGGACGACGGCAACTACAAGACCCGCGCCGAGGTGAAGTTCGAGGGCGACACCCTGGTG
AACCGCATCGAGCTGAAGGGCATCGACTTCAAGGAGGACGGCAACATCCTGGGGCACAAGCTGGAGT
ACAACTACAACAGCCACAACGTCTATATCATGGCCGACAAGCAGAAGAACGGCATCAAGGTGAACTT
CAAGATCCGCCACAACATCGAGGACGGCAGCGTGCAGCTCGCCGACCACTACCAGCAGAACACCCCC
ATCGGCGACGGCCCCGTGCTGCTGCCCGACAACCACTACCTGAGCTACCAGTCCAAGCTGAGCAAAGA
CCCCAACGAGAAGCGCGATCACATGGTCCTGCTGGAGTTCGTGACCGCCGCCGGGATCACTCTCGGCA
TGGACGAGCTGTACAAG

> KLR_MKP1 MKP1 cds in purple, NLS-NES sequence in grey, mNeonGreen cds in green.
TGGAATTCGGCATGGTTGGTCGAGAGGATGCCATGGGAAATGACGAGGCTCCACCAGGTTCTAAAAA
AATGTTTTGGAGAAGCGCGAGCTGGTCAGCATCCCGTACCGCTAGTCAGGTGCCTGAAGGAGACGAAC
AATCCCTTAACATTCCATGCGCGATATCATCAGGGCCATCCAGAAGATGTCCTGCCGCTCCATTAACAC
CGAGATCCCACCACAACTCTAAAGCCAGAGCGTGCCTGCCCCCATTACAACCTCTCGCCATCTCACGA
CGAAGCCTAGATGAGTGGCCAAAAGCCGGGTCAAAGAGGTCAGGCTTGGAAGACCCAGCCACACCAT
CCAAGAAGCCCCGTACTCCCTCTGTTTCTAGTAGACTGGAGCGACTCACACTGCAAAGTTCATTTCAGT
TCCCTTCAGGTACCATGGTCAGTAAAGGGGAGGAAGATAACATGGCATCACTGCCGGCAACACACGA

ACTACACATCTTCGGTTCTATAAATGGGGTTGATTTTGATATGGTTGGACAGGGTACTGGAAATCCGA
ATGATGGATATGAAGAATTGAATTTAAAGAGTACGAAGGGCGATCTTCAATTCTCCCCATGGATCCTC
GTACCCCATATTGGGTACGGCTTCCATCAGTATCTGCCCTATCCAGACGGCATGTCACCTTTCCAGGCC
GCAATGGTAGATGGATCCGGTTACCAAGTACATAGAACGATGCAGTTCGAAGACGGCGCTTCCTTGAC
GGTGAACTATAGGTACACTTACGAAGGAAGCCATATTAAAGGGGAGGCGCAGGTAAAGGGCACTGGG
TTTCCGGCAGATGGACCAGTTATGACGAACTCTCTCACAGCCGCTGATTGGTGCAGGTCAAAGAAGAC
ATACCCCAACGATAAAACAATTATCAGTACGTTCAAGTGGTCATACACCACCGGAAACGGAAAACGA
TATAGGAGTACGGCTAGAACTACATACACATTTGCTAAACCTATGGCTGCAAACTACCTTAAAAACCA
GCCTATGTATGTATTTCGAAAGACAGAATTAAAACACAGTAAAACTGAACTGAATTTTAAGGAGTGGC
AAAAGGCCTTTACAGACGTGATGGGGATGGATGAACTCTATAAA

>KLR_AP2C1 AP2C1 cds in purple, NLS-NES sequence in grey, mNeonGreen cds in green.
CCTCCGGGAGGTGTTTTAAAGAGGAAACGACCTACAAGGCTTGATATACCGATTGGTGTTGCTGGATT
TGTAGCTCCGATTTCTTCATCGGCCGCTGTGGCTGCGAAGAGGTCAGGCTTGGAAGACCCAGCCACAC
CATCCAAGAAGCCCCGTACTCCCTCTGTTTCTAGTAGACTGGAGCGACTCACACTGCAAAGTTCATTTC
AGTTCCCTTCAGGTACCATGGTCAGTAAAGGGGAGGAAGATAACATGGCATCACTGCCGGCAACACA
CGAACTACACATCTTCGGTTCTATAAATGGGGTTGATTTTGATATGGTTGGACAGGGTACTGGAAATCC
GAATGATGGATATGAAGAATTGAATTTAAAGAGTACGAAGGGCGATCTTCAATTCTCCCCATGGATCC
TCGTACCCCATATTGGGTACGGCTTCCATCAGTATCTGCCCTATCCAGACGGCATGTCACCTTTCCAGG
CCGCAATGGTAGATGGATCCGGTTACCAAGTACATAGAACGATGCAGTTCGAAGACGGCGCTTCCTTG
ACGGTGAACTATAGGTACACTTACGAAGGAAGCCATATTAAAGGGGAGGCGCAGGTAAAGGGCACTG
GGTTTCCGGCAGATGGACCAGTTATGACGAACTCTCTCACAGCCGCTGATTGGTGCAGGTCAAAGAAG
ACATACCCCAACGATAAAACAATTATCAGTACGTTCAAGTGGTCATACACCACCGGAAACGGAAAAC
GATATAGGAGTACGGCTAGAACTACATACACATTTGCTAAACCTATGGCTGCAAACTACCTTAAAAAC
CAGCCTATGTATGTATTTCGAAAGACAGAATTAAAACACAGTAAAACTGAACTGAATTTTAAGGAGTG
GCAAAAGGCCTTTACAGACGTGATGGGGATGGATGAACTCTATAAA
